# One month of hyperglycemia alters spectral responses of the zebrafish photopic electroretinogram

**DOI:** 10.1242/dmm.035220

**Published:** 2018-10-22

**Authors:** Zaid Tanvir, Ralph F. Nelson, Kathleen DeCicco-Skinner, Victoria P. Connaughton

**Affiliations:** 1 Department of Biology, American University, 4400 Massachusetts Ave NW, Washington, DC 20016, USA; 2 Neural Circuitry Unit, National Institute of Neurological Disorders and Stroke, National Institutes of Health, 5625 Fisher's Lane, Rockville, MD 20852, USA

**Keywords:** ON bipolar, Outer retina, Diabetes, B-wave, Glucose, A-wave

## Abstract

Prolonged hyperglycemia can alter retinal function, ultimately resulting in blindness. Adult zebrafish adults exposed to alternating conditions of 2% glucose/0% glucose display a 3× increase in blood sugar levels. After 4 weeks of treatment, electroretinograms (ERGs) were recorded from isolated, perfused, *in vitro* eyecups. Control animals were exposed to alternating 2% mannitol/0% mannitol (osmotic control) or to alternating water (0% glucose/0% glucose; handling control). Two types of ERGs were recorded: (1) native ERGs measured using white-light stimuli and medium without synaptic blockers; and (2) spectral ERGs measured with an AMPA/kainate receptor antagonist, isolating photoreceptor-to-ON-bipolar-cell synapses, and a spectral protocol that separated red (R), green (G), blue (B) and UV cone signals. Retinas were evaluated for changes in layer thickness and for the inflammatory markers GFAP and Nf-κB (RelA or p65). In native ERGs, hyperglycemic b- and d-waves were lower in amplitude than the b- and d-waves of mannitol controls. Alteration of waveshape became severe, with b-waves becoming more transient and ERG responses showing more PIII-like (a-wave) characteristics. For spectral ERGs, waveshape appeared similar in all treatment groups. However, a1- and b2-wave implicit times were significantly longer, and amplitudes were significantly reduced, in response to hyperglycemic treatment, owing to the functional reduction in signals from R, G and B cones. Nf-κB increased significantly in hyperglycemic retinas, but the increase in GFAP was not significant and retinal layer thickness was unaffected. Thus, prolonged hyperglycemia triggers an inflammatory response and functional deficits localized to specific cone types, indicating the rapid onset of neural complications in the zebrafish model of diabetic retinopathy.

## Abstract (precis)

**Summary:** Zebrafish can be used to examine diabetic complications, including vision loss. Here, in zebrafish, we show that prolonged (4 week) hyperglycemia causes an inflammatory response associated with functional deficits localized to specific cone types.

## INTRODUCTION

Diabetic retinopathy (DR) is a leading cause of blindness and visual impairment in the United States (nei.nih.gov/health/diabetic). DR occurs in individuals with long-standing diabetes mellitus, affecting 40-50% of all patients (Arden and Ramsey, 2015). Retinal complications in DR are believed to be caused by prolonged high levels of glucose (hyperglycemia) that increase inflammatory mediators (Kern, 2007; Romeo et al., 2002) and vascular permeability (Guthrie and Guthrie, 2004). Increased permeability stimulates the formation of new, fragile vessels that can subsequently rupture. The resulting hemorrhage and ischemia damages retinal nerve cells, resulting in vision loss.

In neural retina, reactive gliosis in Müller glia, characterized by an increase in glial fibrillary acidic protein (GFAP) levels, is observed in human diabetic retinas (Carrasco et al., 2008) and in animal models (Agardh et al., 2001; Barber et al., 2000; Rungger-Brandle et al., 2000; Zeng et al., 2000). Nuclear factor-κB (Nf-κB; RelA or p65) levels also increase, serving as a pro-apoptotic signal to pericytes (Kowluru and Chan, 2007; Patel and Santani, 2009). Clinically, multifocal (Xu et al., 2006) or full-field electroretinograms (ERGs) are used to determine whether retinal function is compromised in DR (Tzekov and Arden, 1999). Full-field ERGs identify photoreceptor (a-wave), ON bipolar (b-wave) and amacrine cell responses (oscillatory potentials, OPs) (Tzekov and Arden, 1999). When a long flash of light is used, the d-wave (OFF bipolar) component is also revealed (Lung et al., 2016; Sieving, 1993). Diabetic patients are reported to have altered a-wave, b-wave and OPs, with the degree of change correlated with the severity of DR. Decreased and delayed OPs are consistently observed in the early stages of DR. Decreased b-wave amplitudes also occur early (Kizawa et al., 2006), whereas changes in the a-wave component are not observed until more advanced stages (Kizawa et al., 2006; Tzekov and Arden, 1999). Decreased b-wave amplitude, smaller/slower OPs (Bui et al., 2009; Hancock and Kraft, 2004; Layton et al., 2007; Li et al., 2002) and reduced OFF responses (Samuels et al., 2015, 2012) are observed in animal models of DR, with more variable changes in the a-wave component (Bui et al., 2009; Hancock and Kraft, 2004; Kohzaki et al., 2008; Li et al., 2002; Phipps et al., 2006). Disruptions of A17 amacrine feedback onto rod bipolar axon terminals in the inner retina have been reported (Castilho et al., 2015).

Zebrafish (*Danio rerio*) are a new model organism in which to examine complications associated with prolonged high blood sugar levels. Zebrafish have an insulin-secreting pancreas (Argenton et al., 1999) and genes related to diabetes – such as those encoding insulin, IA-2 autoantigen and IA-2β autoantigen – have been cloned (Rubenstein, 2003). Although zebrafish retinal vessels are restricted to the inner limiting membrane (Alvarez et al., 2007; Chapman et al., 2009), they are in direct contact with Müller cell endfeet (Alvarez et al., 2007), and vascular endothelial cells are connected via junctional complexes and surrounded by pericytes (Alvarez et al., 2007). In response to hyperglycemic conditions, induced by glucose exposure in the water (Alvarez et al., 2010; Capiotti et al., 2014; Connaughton et al., 2016; Gleeson et al., 2007) or streptozotocin injections (Intine et al., 2013; Olsen et al., 2010), the inner retina thins (Gleeson et al., 2007; Olsen et al., 2010), the basement membranes of retinal vessels thicken, cone morphology is altered (Alvarez et al., 2010) and glycated proteins are formed (Capiotti et al., 2014). These are characteristics observed in other animal models as well as in humans.

Our laboratory developed an alternating immersion protocol that induces and maintains hyperglycemic conditions in zebrafish (Connaughton et al., 2016; Gleeson et al., 2007), mimicking the oscillations in blood glucose levels seen in humans with poorly controlled diabetes (Ceriello et al., 2008). Subsequent use of this technique by other investigators (Alvarez et al., 2010) identified physiological changes in glucose-treated animals. These functional changes were identified using a white-light stimulus and correlated with aberrant cone morphology (Alvarez et al., 2010). To determine whether these functional changes are caused by alterations in specific cone photoreceptor type(s), we recorded photopic ERG responses to white light and spectrally distinct light stimuli over a range of intensity levels in a superfused zebrafish eyecup preparation. In the spectral stimulation protocol, the AMPA/kainate (KA) receptor blocker 6-cyano-7-nitroquinoxaline-2,3-dione (CNQX) was added to the medium. This restricted ERG a- and b-wave signals to the outer retina, as only cones and ON bipolar cells respond in the presence of the blocker. In the white-light protocol, the medium contained no blockers, so that both the inner and outer retina circuits were functional. A major goal was to identify changes in spectral sensitivity owing to 1 month of glucose exposure and, if present, to identify the response of the cone type(s) that might be most susceptible. A further goal was to identify contributions of the inner retina to hyperglycemia-induced dysfunction.

## RESULTS

**Fig. 1.**
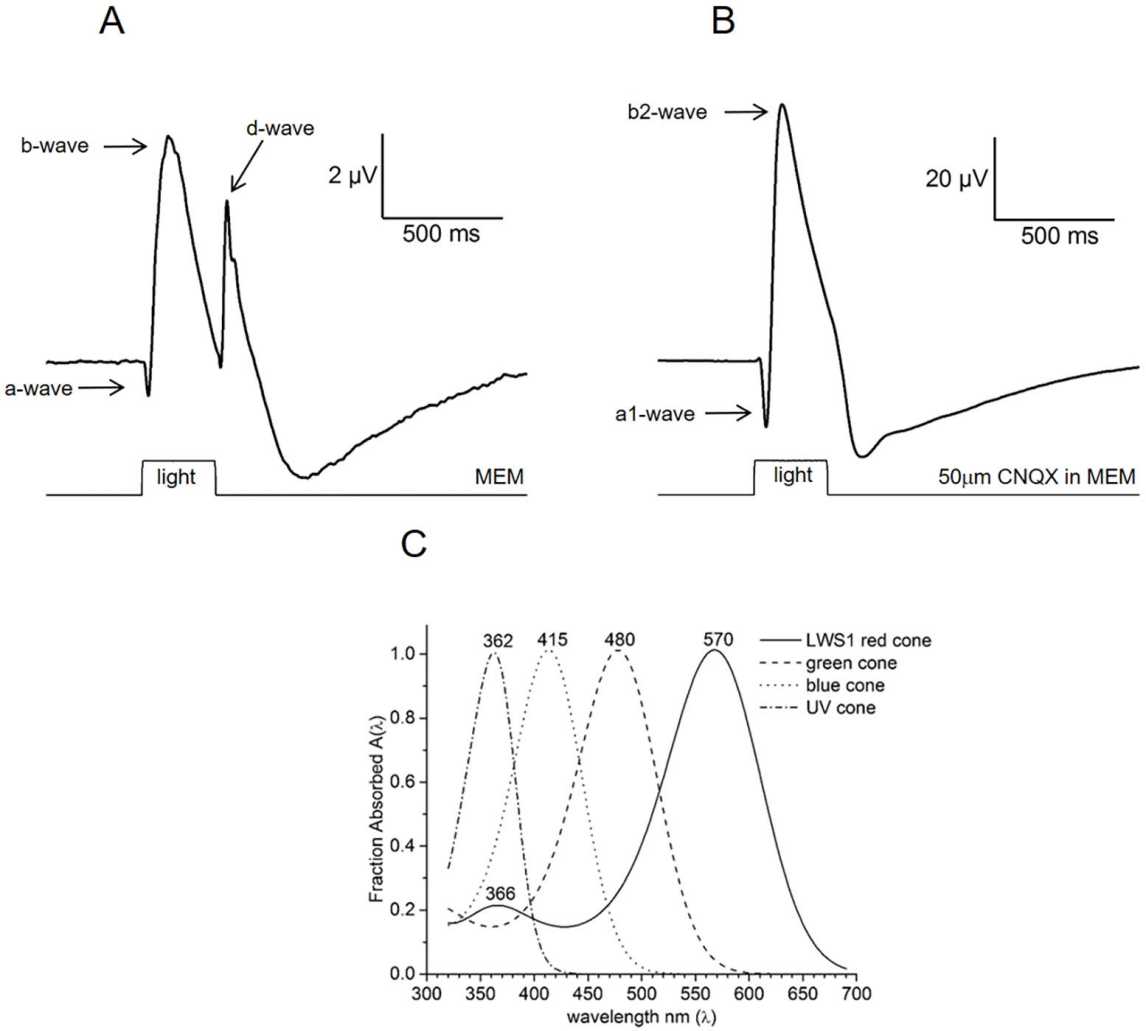
**Mean ERG waveforms in native and spectral datasets.** (A) Native ERG is the mean response to 70 white-light stimuli (Xenon) covering a 3.5 log unit range in brightness in a water-treated control eyecup. The eyecup perfusate does not contain CNQX. In the absence of blocker, native a-, b- and d-waves are evident. (B) Mean spectral ERG trace in the presence of 50 µM CNQX from a water-treated control retina (blue background) showing the a1-wave and b2-wave components. This mean is from 70 stimuli of wavelengths between 330 nm and 650 nm, covering a 3.5 log unit range in brightness at each wavelength. The AMPA/KA receptor blocker limits the ERG components to those arising from cones and ON-type bipolar cells. CNQX induces an increase in a- and b-wave amplitudes. In A and B, the stimulus ranges were adjusted to evoke responses from threshold to near saturation. The examples are from individual treated eyes. (C) Opsin absorbance spectra of UV (362 nm), blue (415 nm), green (480 nm) and red (570 nm) zebrafish cone types. The 366 nm secondary peak is the R cone beta band. These absorbance spectra are integrated into the spectral model used to extract individual cone component responses from the massed signal of the ERG across wavelengths. R, G and B cone spectra are from Hughes et al. (1998); the UV spectrum is from Palacios et al. (1996).

As previously reported (Nelson and Singla, 2009), light stimulation of isolated zebrafish eyecups superfused in medium containing an AMPA/KA receptor antagonist resulted in a negative-going wave, the a1 component of the a-wave, immediately after light ON, followed by a large, positive wave, the b2 component of the b-wave. Recordings evoked in response to a white-light stimulus, without CNQX in the bath, included a-wave, b-wave and d-wave components (Fig. 1A,B). Individual ERG responses similar to these were analyzed individually and by means of the spectral model, to determine treatment-induced changes in cone sensitivities and overall retinal responses. Morphological analyses of thick sections and changes in GFAP and/or Nf-κB content were correlated with the physiological data.

### Blood sugar and wet weight measurements

Blood sugar measurements of fish alternately exposed to 2% glucose solution/0% glucose solution averaged 135±36.7 mg/dL (mean±s.e.m.; *n*=7 fish) after 28 days of treatment, a significant increase compared with values from mannitol-treated (*n*=8) and water-treated (*n*=6) controls (Table 1A; one-way ANOVA, *P*=0.011). Wet weights of these same fish were not significantly different (Table 1B; one-way ANOVA, *P*=0.59). These changes in blood sugar, but not wet weight, are consistent with previous reports using an alternating 2% glucose solution/0% glucose solution for 1 month, with fish <1 year of age (Connaughton et al., 2016).

**Table 1.**
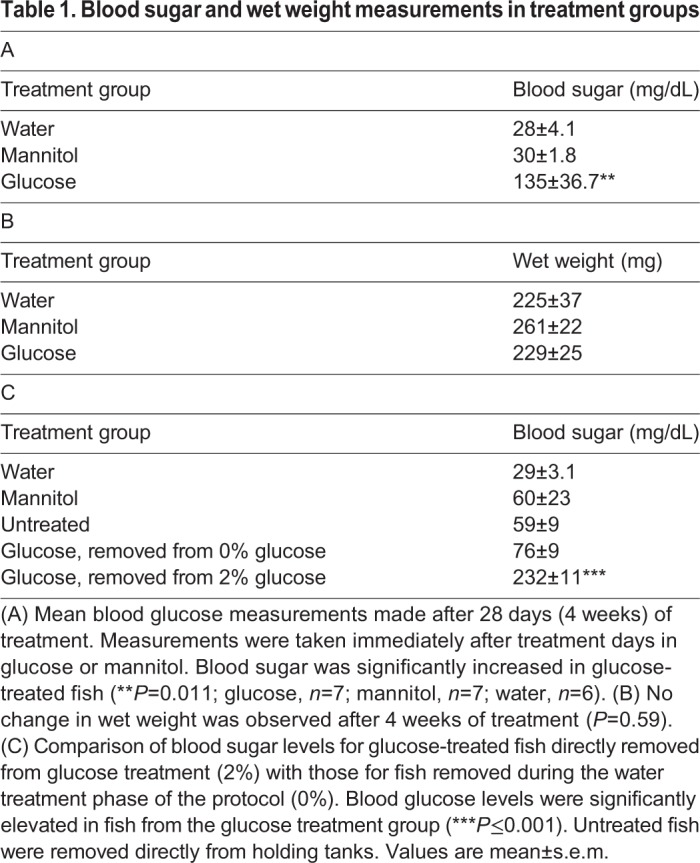
**Blood sugar and wet weight measurements in treatment groups**

Once ERG recordings began, 2 fish per day were removed from the treatment. This resulted in some fish remaining in treatment for longer than 28 days. To confirm that these fish were still hyperglycemic, blood sugar measurements were collected at the time of ERG recordings (Table 1C). In glucose-treated fish directly removed from 2% glucose, blood glucose averaged 232±10.74 mg/dL (*n*=3); in glucose-treated fish removed from water (0% glucose), blood glucose averaged 76±9 mg/dL (*n*=2). The elevated blood sugar measurements from glucose-exposed fish directly removed from glucose were significantly different (one-way ANOVA, *P*<0.001) from all other values, including those from water-treated controls (*n*=4, outlier removed), mannitol-treated controls (*n*=4) and untreated fish directly removed from stock tanks (*n*=3) (Table 1C).

### White-light stimuli

**Fig. 2.**
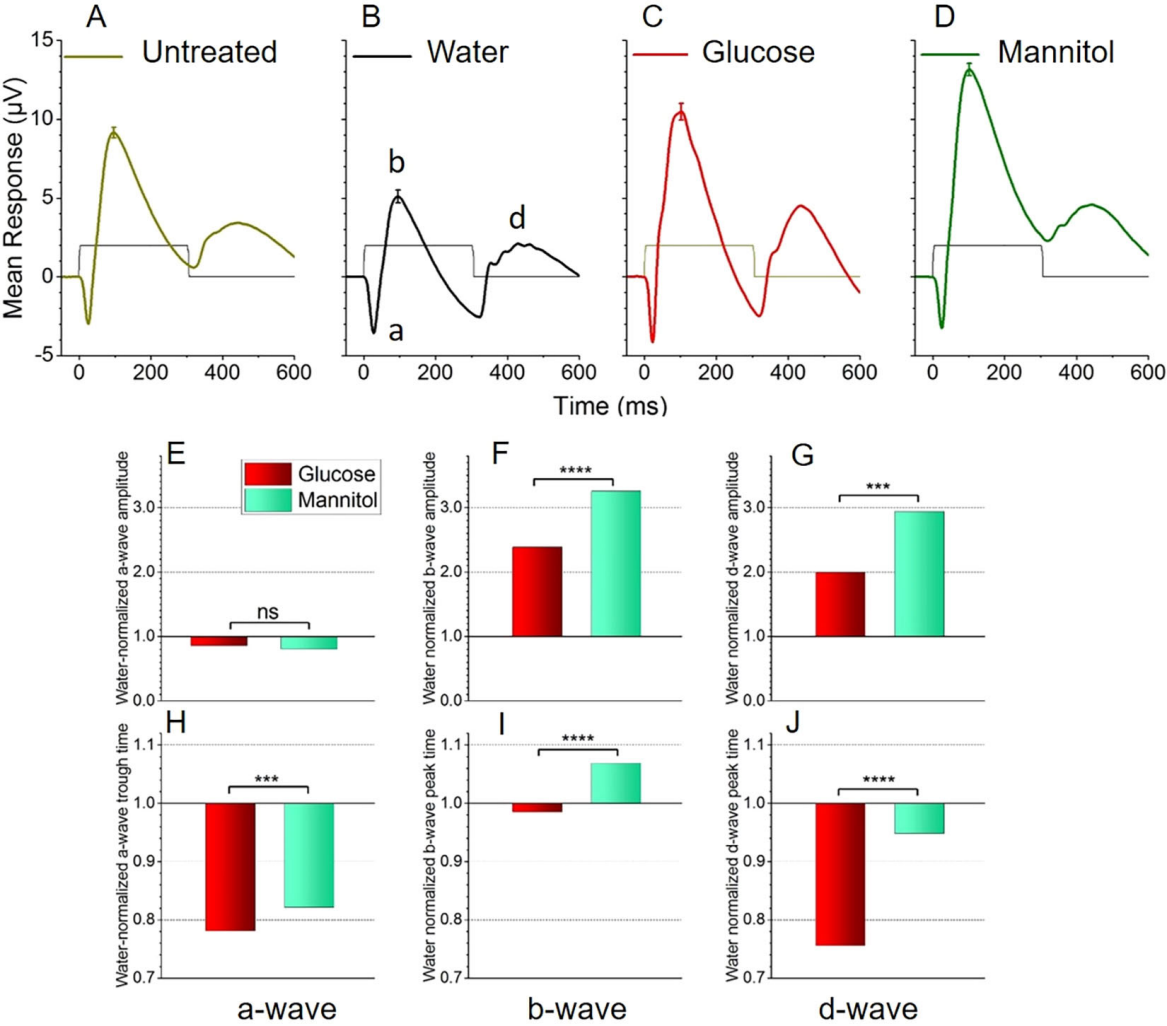
**Changes in glucose versus mannitol native ERG parameters with white-light stimulus.** Under ‘native ERG’ conditions, white-light stimuli evoked a-, b- and d-waves in all treatments (a-, b- and d-waves are labeled in B). (A-D) Mean ERG response from untreated fish removed directly from stock tanks (A), and water-treated (B), glucose-treated (C) and mannitol-treated (D) fish. Mean waveforms differ among treatment groups and a handling stress – identified by the reduced values in water-treated controls – was observed. Glucose- and mannitol-treated responses were subsequently normalized to water control values to account for this handling stress. All stimuli are white light (Xenon) attenuated by 3 log units on an IR background, and evoked near-saturated amplitudes. Untreated (A), glucose (C) and mannitol (D) waveforms reflect the mean of 300 responses from 10 eyes; the water (B) waveform is a mean of 210 responses from 7 eyes. The error bars at the b-wave peaks are s.e.m. The rectangular trace shows the 300 ms light stimulus. (E-J) Mean ERG parameters from the response traces in A-D normalized to water control values and compared. Glucose treatment increased the a-wave amplitude, but not significantly (E). In contrast, b-wave and d-wave amplitudes were significantly reduced in glucose-treated retinas compared with mannitol-treated retinas (F,G). Glucose treatment also quickened a-wave (H), b-wave (I) and d-wave (J) implicit times (time-to-peak). The water-treated mean control values were as follows: (E) −2.95±0.20 µV, *n*=211; (F) 3.19±0.40 µV, *n*=211; (G) 1.27±0.23 µV, *n*=211; (H) 28.9±0.46 ms, *n*=189; (I) 98.9±1.21 ms, *n*=188; (J) 150.8±5.15 ms, *n*=210. Asterisks use GraphPad significance convention for Student's *t*-tests (ns, *P*>0.05; ***P≤0.001; ****P≤0.0001).

To be consistent with published reports (Alvarez et al., 2010), we recorded ERGs using a white-light stimulus at saturating stimulus intensities, without AMPA/KA receptor blockade. Under these conditions, mean ERG responses with clear a-wave, b-wave and d-wave components were evoked in all treatment groups (Fig. 2A-D). These data revealed that the response of water-treated fish (Fig. 2B) was consistently reduced compared with that of untreated fish (Fig. 2A), reflecting a handling stress. Consequently, we normalized responses from glucose- and mannitol-treated tissue to those of the water-treated controls (see Materials and Methods).

Mean b-wave amplitudes (µV) modeled from 300 responses in 10 glucose-treated eyes (7.6±0.5 µV) at the brightest stimulus intensity were significantly decreased (*P*<0.001), compared with average b-wave amplitudes from the same number of responses in mannitol-treated eyes (10.4±0.3 µV, Fig. 2F); d-wave amplitudes were also significantly decreased in glucose-treated compared with mannitol-treated eyes (*P*<0.001, Fig. 2G). In contrast, there was no difference in mean peak a-wave amplitude across the groups (Fig. 2E). Similar trends were observed when the analysis was performed combining responses from all 7 light intensities (Fig. S1A-C), except for a-wave amplitude, which was significantly increased (*P*<0.001) in the cumulative dataset.

At the brightest stimulus intensity, mean peak implicit times (i.e. time-to-peak) for all ERG components were significantly shorter (*P*<0.001) in glucose-treated tissue (Fig. 2H-J; *n*=278-287 responses from 10 glucose eyes; *n*=300 responses from 10 mannitol eyes). Specifically, b-wave time averaged 97.5±1.08 ms in glucose-treated eyes, compared with an average of 105.8±19.8 ms in mannitol-treated tissue; and d-wave implicit time in glucose-treated tissue averaged 114.0±3.6 ms, versus 143.0±3.7 ms in mannitol-treated retinas. Similarly, the a-wave glucose-treated implicit time of 22.6±0.29 ms was less than the average time (23.7±0.17 ms) in the mannitol-treated group. Analysis of implicit times for all light intensities revealed the same results, and is provided in Fig. S1D-F.

### Stimulating eyecups with different spectral wavelengths

To determine whether the observed changes in the native ERG (Fig. 2) after 1 month of alternating glucose immersion were caused by differential sensitivity of the different cone signals in the outer plexiform layer (OPL), we looked at responses to 4 of the 9 stimulus wavelengths – 570 nm, 490 nm, 410 nm and 370 nm – in detail. These wavelengths are close to peak opsin absorbance for adult zebrafish red (R), green (G), blue (B) and UV cones, respectively (Robinson et al., 1993). Whereas R cones might be expected to respond to all these stimuli, the shorter wavelength cones would not be expected to respond to stimuli with wavelengths significantly longer than their absorbance peaks. The ability of the wavelength filters to selectively stimulate cone types is shown in Table S1.

**Fig. 3.**
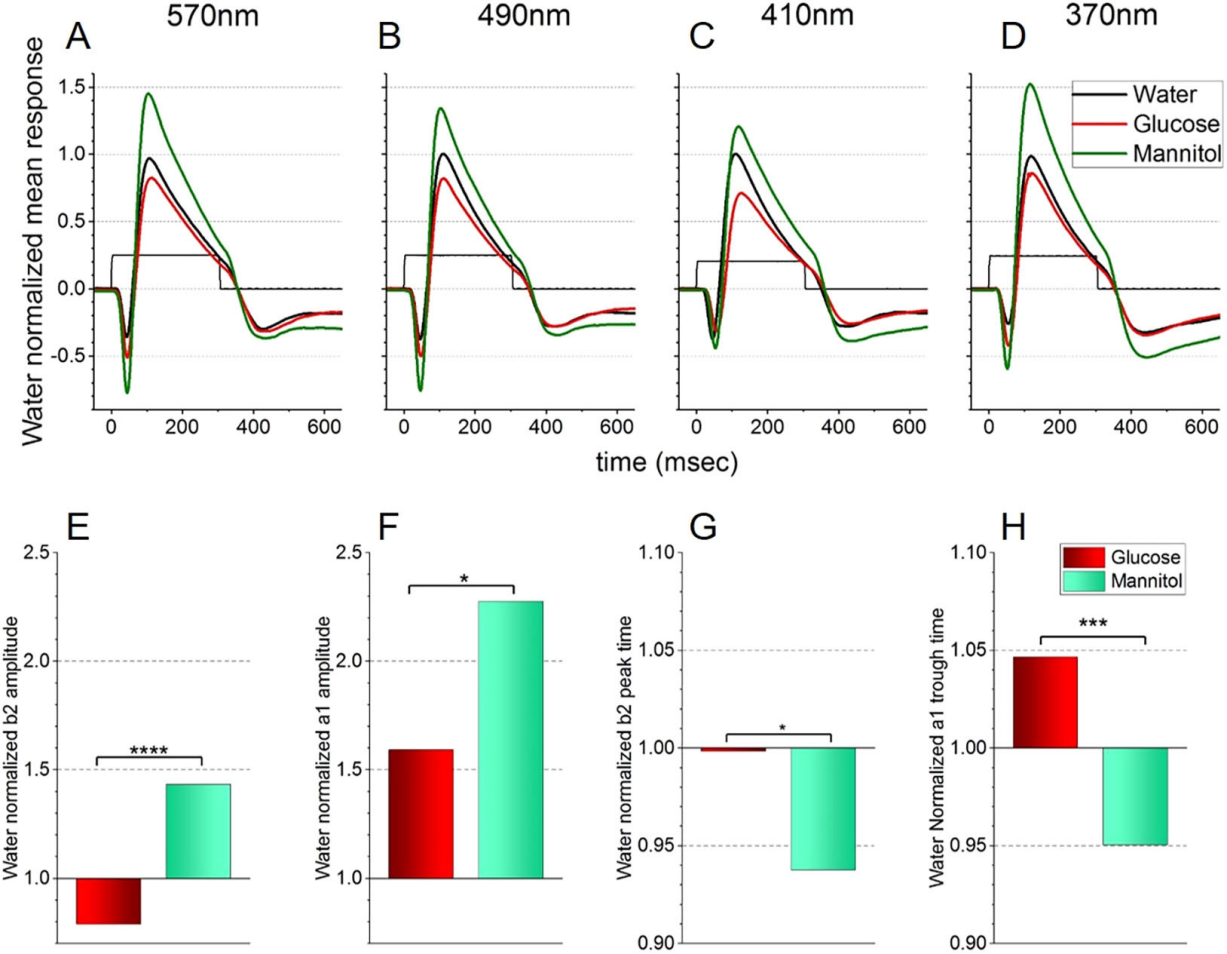
**Treatment effects on spectral ERG signals from outer retina (blue background).** Responses are recorded on a blue (418 nm) background that selectively suppressed B and G cones. Distal retina b2-wave and a1-wave amplitudes are isolated with an AMPA/KA receptor antagonist (CNQX, 50 µM), which isolates signals from cones and from ON bipolar cell synapses. (A-D) Mean waveforms to saturating monochromatic stimulation near the absorbance peaks for R cones (A), G cones (B), B cones (C) and UV cones (D). For each wavelength the irradiance is a 2 log unit attenuation of the beam, with energies ranging from 5.4 log(hν µm^−2^ s^−1^) at 370 nm to 6.0 log(hν µm^−2^ s^−1^) at 490 nm. (E-H) Mean normalized response amplitudes for b2-wave (E) and a1-wave (F) and mean time-to-peak for b2-wave (G) and a1-wave (H). Glucose, *n*=104 responses from 13 eyes; mannitol, *n*=100 responses from 15 eyes; water, *n*=84 responses from 13 eyes. With these stimuli, glucose-treated fish show reduction in overall mean a1 and b2 amplitudes and longer implicit times. Data from all intensities are shown in Fig. S3A-D. Asterisks denote significant differences (Student's *t*-tests, GraphPad asterisk convention; *P≤0.05; ***P≤0.001; ****P≤0.0001). The water control values (from 84 responses, 13 eyes) used for normalization were as follows: a1 amplitude, 4.5±0.4 µV; b2 amplitude, 22.0±2.2 µV; a1 peak time, 52.7±1.3 ms; b2 peak time, 118.5±1.8 ms.

In recordings using the blue (418 nm)-adapting background, AMPA/KA receptor blocker and near-saturating chromatic stimuli, mean ERG timecourses look similar in shape across the 4 stimulating wavelengths (Fig. 3A-D), although mean peak a1 and b2 amplitudes appear reduced in glucose-treated eyes (104 responses from 13 eyes), compared with mannitol-treated eyes (100 responses from 15 eyes). These observations were confirmed statistically under 4-wavelength, saturating-irradiance stimulus conditions (Fig. 3E-H). The normalized b2 mean amplitude in glucose-treated eyes (17.4±1.4 µV) was significantly smaller (*P*<0.001, Fig. 3E) than the mean value from mannitol-treated eyes (31.5±2.1 µV). The mean a2 amplitude in glucose-treated eyes (7.22±0.56 µV) was also reduced compared with that of the mannitol-treated group (10.3±1.1 µV, *P*<0.05, Fig. 3F).

There were small, but significant, variations in implicit times. The glucose-treated b2-wave time (118.3±24.3 ms) was longer than the mannitol-treated value (111.1±1.8 ms, *P*<0.05, Fig. 3G), as was the implicit time for the a1 component (glucose average, 55.2±1.1 ms; mannitol average, 50.1±0.8 ms) (*P*<0.001, Fig. 3H). Analyzing responses averaged across all wavelengths and brightnesses, as shown in Fig. S2A-D, resulted in the same amplitude reductions.

**Fig. 4.**
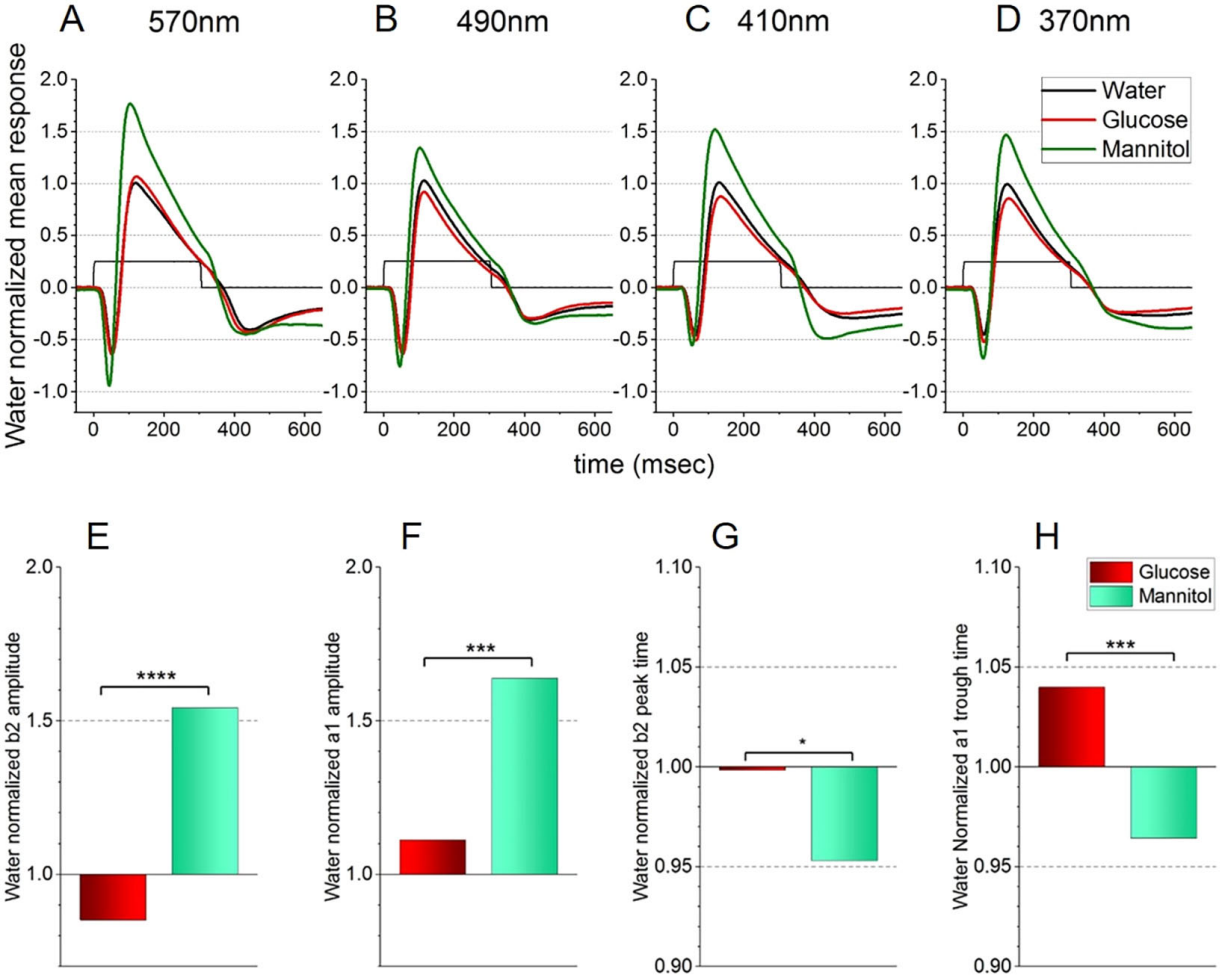
**Treatment effects on spectral ERG signals from the outer retina (red background).** Responses are recorded on a red (627 nm) background. b2-wave and a1-waves are isolated with an AMPA/KA receptor antagonist (CNQX, 50 µM), which blocks horizontal cell, OFF bipolar cell and inner retina responses. (A-D) Responses to saturating monochromatic stimulation near the absorbance peaks for R cones (A), G cones (B), B cones (C) and UV cones (D). For each wavelength, the irradiance is a 2 log unit attenuation of the beam, with energies ranging from 5.4 log(hν µm^−2^ s^−1^) at 370 nm to 6.0 log(hν µm^−2^ s^−1^) at 490 nm. (E-H) Mean normalized response amplitudes for b2-wave (E) and a1-wave (F), and mean time-to-peak for b2-wave (G) and a1-wave (H). Glucose, *n*=112 responses (14 eyes); mannitol, *n*=108 responses (14 eyes); water, *n*=104 responses (14 eyes). At these stimulating wavelengths, glucose-treated fish show reduced a1 and b2 amplitudes compared with those of mannitol-treated fish, and longer implicit times. Data from all intensities are shown in Fig. S3E-H. The water control values used (from 104 responses, 14 eyes) were as follows: a1 amplitude, 7.5±0.5 µV; b2 amplitude, 18.3±1.6 µV; a1 peak time, 57.8±0.7 ms; b2 peak time, 125.9±1.8 ms (Student's *t*-test, GraphPad asterisk convention; *P≤0.05; ***P≤0.001; ****P≤0.0001).

For the red (627 nm) background and AMPA/KA receptor blocker, the mean ERG timecourses in the different treatment groups were similar for near-saturating 570 nm, 490 nm, 410 nm and 370 nm stimuli (Fig. 4A-D). The analyzed data (Fig. 4E-H) are based on 112 responses in glucose-treated eyes and 108 responses in mannitol-treated eyes under the 4-wavelength, saturating stimulus conditions illustrated (Fig. 4E-H). As in Fig. 3, peak b2 and a1 amplitudes were reduced in glucose-treated, compared with mannitol-treated, eyes. This was confirmed statistically as the mean b2 amplitude in glucose-treated eyes was 15.6±1.3 µV, a value found to be significantly smaller than the mean value in mannitol-treated controls (28.0±1.6 µV, *P*<0.0001). The mean a2 amplitude in glucose-treated eyes (8.4±0.6 µV) was also significantly reduced compared with that of mannitol controls (12.1±0.8 µV, *P*<0.001). As found with the blue-adapting background, both the mean glucose-treated b2 peak time (125.7±1.9 ms) and a1 peak time (60.1±0.9 ms) were significantly longer than the times from mannitol-treated tissue (b2, *P*<0.01; a1, *P*<0.001). The same amplitude reductions were also obtained in the responses to all brightnesses and wavelengths in the cumulative dataset (Fig. S2E-H).

Taken together, the finding that glucose decreases b2 and a1 amplitudes at all wavelengths on blue backgrounds certainly suggests the involvement of R cones, the only cones that can be stimulated by all wavelengths. The fact that this pattern of amplitude decrease also persists on red backgrounds suggests that at least one other cone could also be involved. These results further suggest that reductions in native ERG b- and d-wave amplitudes, if not implicit times, might originate in distal retina.

### Spectral analysis

**Fig. 5.**
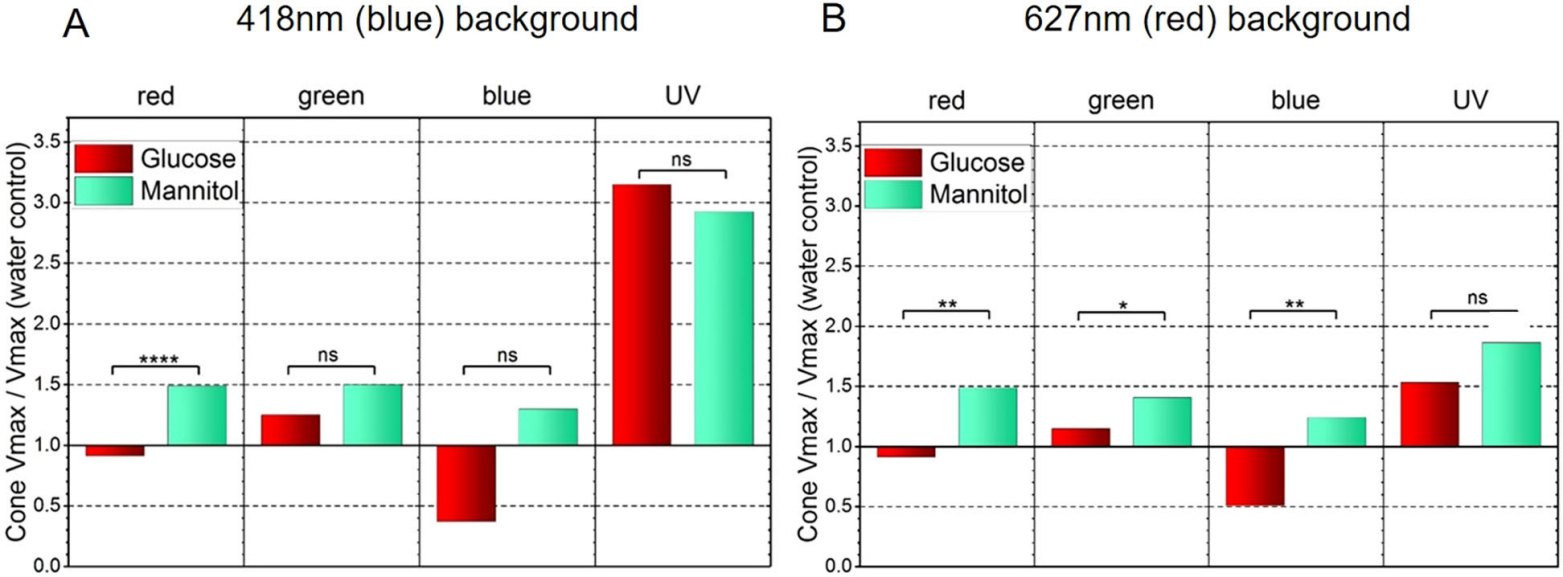
**Spectral ERG model identifies changes in peak signals of cone types.** R, G, B and UV cone signal amplitudes in the ERG b2-wave for glucose and mannitol groups are extracted using the spectral analysis model (Eqn 1) and normalized to values in the water control. (A) On a blue (418 nm) background, glucose treatments reduce R cone saturation amplitude compared with mannitol treatments. (B) A red 627 nm background identified reduced R cone peak amplitudes in glucose-treated tissue. Additionally, peak amplitude reduction was seen in G and B cones, with respect to mannitol. These ERG b2 signals are b-waves measured under AMPA/KA receptor blockade (50 µM CNQX) and contain only cone and ON bipolar cell signals from the outer retina (Student's *t*-test, GraphPad asterisk convention; *P≤0.05, **P≤0.01, ****P≤0.0001; ns, nonsignificant). Data points fit to Eqn 1: (A) glucose, 1120; mannitol, 1469; water, 630; (B) glucose, 1190; mannitol, 1750; water, 910. Cone amplitudes (µV) in water treatments were as follows: (A) R, 34.0±0.8; G, 25.6±1.7; B, 6.4±1.2; U, 2.2±0.1; (B) R, 26.5±1.2; G, 33.4±1.4; B, 11.0±1.3; U, 4.3±1.0.

To analyze the physiological responses evoked by the different stimulating wavelengths in terms of cone components, the spectral properties of the cumulative spectral dataset within each treatment group were compared by fitting to Eqn 1. With this equation, saturating voltages (Vr, Vg, Vb, Vu) for signals arising from each cone can be determined. Using this approach, we compared outer retina ERG b2 signals, isolated by AMPA/KA receptor blockade, from glucose-treated and mannitol-treated control groups on the blue (418 nm) background (Fig. 5A). A significant reduction in maximal response of the glucose-treated R cone signals (V_r_, Eqn 1) was found (*P*<0.0001), whereas no significant effect on peak amplitudes of G, B or UV cones was detected (green, *P*=0.20; blue, *P*=0.12; UV, *P*=0.87), even though a tendency toward smaller G and B cone signals is apparent. Because the blue background might be selectively desensitizing for B and G cones, the experiment was repeated on a red (627 nm, Fig. 5B) background, which does not desensitize them. Even though R cones are desensitized by this background, a very significant reduction in peak R cone signal amplitude was induced by glucose treatment compared with mannitol treatment (*P*<0.01). The ability to elicit R cone responses with a red-adapting background occurred because R cone amplitudes are not eliminated by red backgrounds. However, their half saturation (kr) does increase.

Significant reductions in G and B cone saturation amplitudes (V_g_, V_b_) were also observed (green, *P*<0.05; blue, *P*<0.01). In contrast, no significant signal amplitude differences were noted for UV cones (*P*=0.58). These findings further confirm that actions on the glucose-mannitol axis occur under AMPA/KA receptor blockade and affect R cone phototransduction and ON bipolar synaptic currents strongly; G and B cone signaling is also affected, although to a lesser extent.

Therefore, the spectral model supports the changes in b2-wave amplitudes observed for individual stimulating wavelengths and indicates that the b2-wave signals arising from R and G (double) cones, and potentially also B cones, are significantly reduced in hyperglycemic, glucose-treated fish.

### Changes in retinal anatomy and neurochemistry

**Fig. 6.**
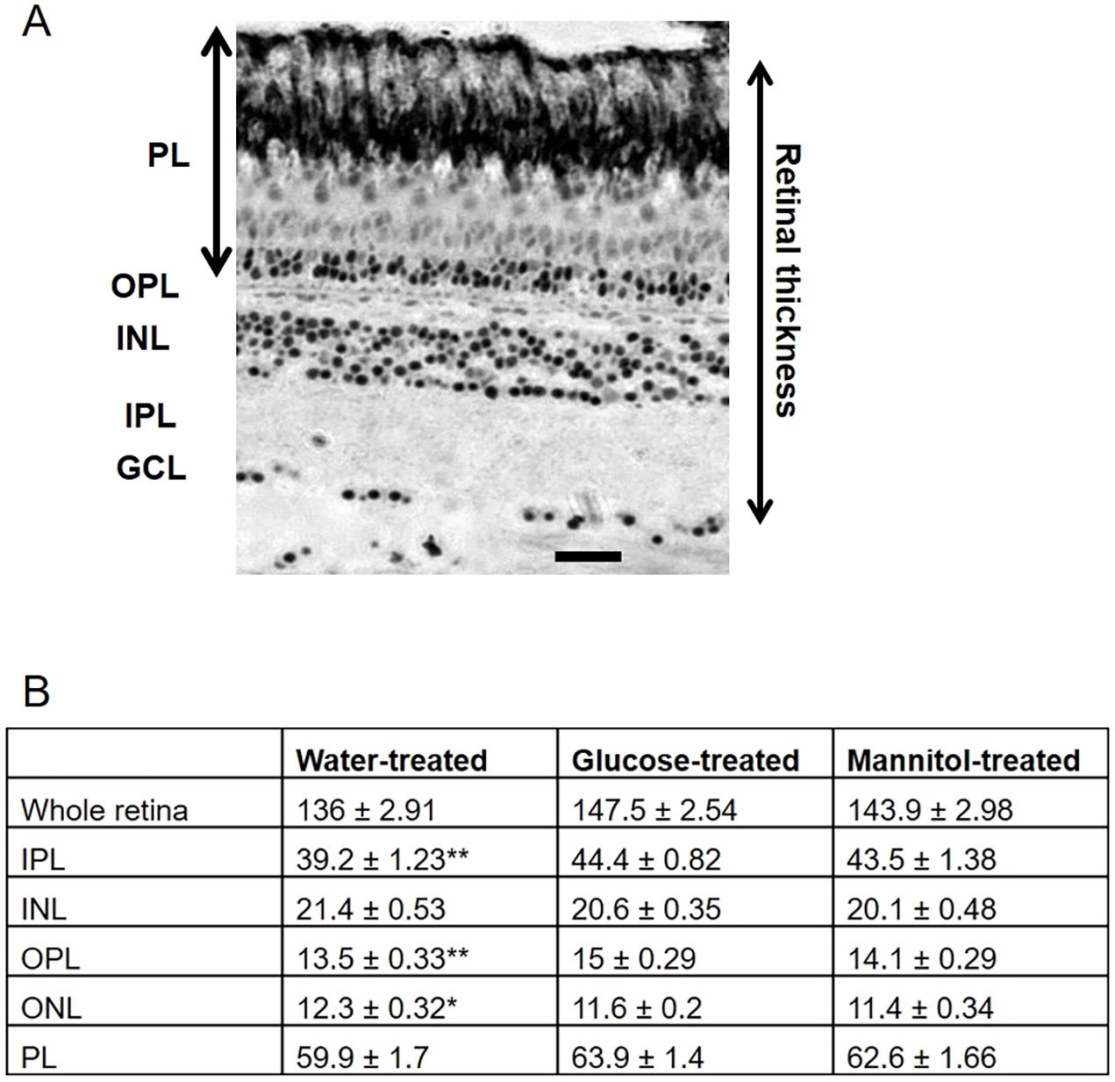
**Mean thickness measurements (±s.e.m.) of retinal layers from tissue exposed to alternating water, glucose or mannitol for 4 weeks.** (A) Representative retinal thick section with retinal layers labeled on the left. Measurements were made of each layer from the ganglion cell layer (GCL) to the distal edge of the photoreceptor layer (PL). INL, inner nuclear layer; IPL, inner plexiform layer; OPL, outer plexiform layer. Scale bar: 20 μm. (B) Significant differences were observed for the IPL (*P*=0.008), OPL (*P*=0.005) and outer nuclear layer (ONL) (*P*=0.048) measurements only. In these cases, changes in layer thickness were observed in water-treated, compared with glucose- and mannitol-treated, tissue; however, no differences were observed between glucose- and mannitol-treated animals (*P≤0.05, **P≤0.01). Units are μm. *n*=7 eyes (water), *n*=10 eyes (glucose), and *n*=7 eyes (mannitol).

Thickness measurements revealed a central retinal thickness of 136 µm (±2.91) in water-treated retinas, a value not significantly different from those of glucose- (147.5±2.54 µm) or mannitol-treated (143.9±2.98 µm) tissue. For some specific layers [inner plexiform layer (IPL), *P*<0.01; OPL, *P*<0.01], however, mean thickness values were larger in glucose and mannitol treatment groups compared with water-treated controls (Fig. 6), whereas outer nuclear layer (ONL) thickness was significantly less in these 2 treatment groups (*P*=0.048). Nonetheless, despite observations of physiological differences in glucose and mannitol groups, no changes in retinal layering structure were seen. The similar retinal thicknesses in both mannitol- and glucose-treated tissue is consistent with published findings using Epon-embedded sections (Alvarez et al., 2010).

**Fig. 7.**
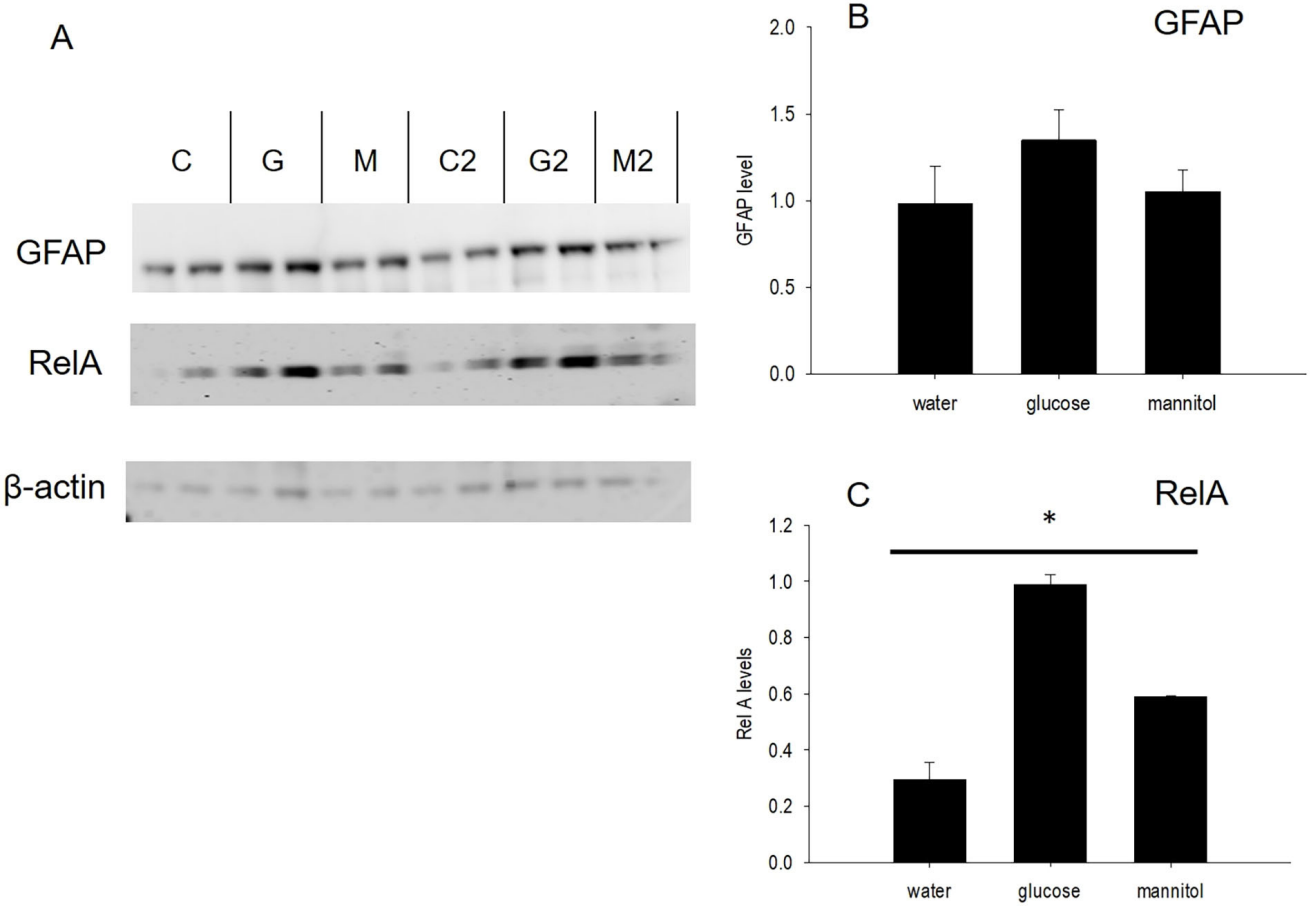
**GFAP and Nf-κB levels increase in glucose-treated retinas.** (A) Western blot probed with antibodies against GFAP (top) and RelA (Nf-κB, middle). β-actin (bottom) served as the loading control. (B,C) Densitometric analysis identified increases in mean GFAP (B) and Nf-κB (C) content in glucose-treated retinal homogenates compared with water-treated or mannitol-treated preparations. C, water-treated control; G, glucose treated; M, mannitol treated. C2, G2 and M2 represent replicates for the C, G and M groups. Error bars represent s.e.m. The asterisk in C indicates that differences in RelA levels were significant across all 3 treatment groups (*P*≤0.003).

Additional analysis of retinal tissue was performed using western blotting, to identify changes in protein content within retinal homogenates. We chose to probe for GFAP, a protein expressed in Müller glia, and the Nf-κB inflammatory marker, which are known to be upregulated by hyperglycemic conditions (Barber et al., 2000; Gerhardinger et al., 2005; Kowluru and Chan, 2007; Patel and Santani, 2009; Romeo et al., 2002). Both proteins were upregulated in glucose-treated zebrafish retinas (Fig. 7). GFAP content was increased 1.4× in homogenates from glucose-treated retinas, although this change was not significant (*P*=0.411) (Fig. 7). However, Nf-κB content (determined using the anti-RelA antibody) was increased in glucose-treated retinas by 3.5× and in mannitol-treated retinas by 2× (Fig. 7). These differences in RelA levels were significant (*P*=0.003).

## DISCUSSION

Zebrafish were suggested as a model for type 2 diabetes when alternate immersion in a glucose solution increased blood glucose values and thinned the inner retina (Gleeson et al., 2007). Subsequent studies showed that alternating glucose immersion also thickened basement membranes in retinal vasculature (Alvarez et al., 2010) and glycated proteins in the eye (Capiotti et al., 2014), both of which are observed in diabetic patients and in other animal models. Zebrafish clearly respond to glucose in water (Alvarez et al., 2010; Capiotti et al., 2014), with uptake of a fluorescently tagged glucose probe into larvae occurring ∼30 min after immersion (Lee et al., 2013). A zebrafish type 1 diabetes model has also been developed in which repeated injection of streptozotocin ablates pancreatic β-cells, leading to elevated blood sugar levels (Intine et al., 2013; Olsen et al., 2010). These studies, along with research showing that the zebrafish pancreas has characteristics similar to those of the mammalian pancreas (Kinkel and Prince, 2009), suggest that the zebrafish is a good model in which to examine the effects of complications associated with chronic high blood sugar, as seen in diabetes.

### Alternate glucose immersion increases blood sugar levels

Here, we examined changes in cone-specific physiological responses resulting from alternating exposure to 2% glucose (∼110 mM) (Alvarez et al., 2010) for 1 month. As previously reported (Connaughton et al., 2016; Gleeson et al., 2007), 1 month of exposure elevated blood sugar values to ∼3× higher than those of water- and mannitol-treated controls. To accommodate physiological testing, we extended glucose treatments to 5 weeks. Blood sugar remained elevated with continued treatment, as fish removed from glucose at the time of ERG recordings continued to display significantly elevated blood sugar levels. Although blood sugar was different across the treatment groups, wet weight and overall survival/behavior were not, consistent with published reports (Connaughton et al., 2016). This indicated that the overall health of the fish was comparable in the different treatment groups.

Whereas the overall health for the water-treated fish was like those in the other treatment groups, the ERG responses were not. b-wave amplitudes recorded from water-treated controls were reduced compared with those of untreated fish (Fig. 2A, *P*<0.0001), suggesting that the daily transfers are stressful to the fish, despite no overt differences in behavior and survival across groups. Handling is a well-known stressor to fish that can affect hormone levels (Acerete et al., 2004; De Lombaert et al., 2017), as well as brain chemistry (Enrico et al., 1998; Timmerman et al., 1999). Our data suggest that handling/daily transfers of the fish can also affect retinal (ERG) responses. Given that the glucose- and mannitol-treated fish were similarly handled/transferred, we normalized our ERG amplitude data to responses from the water-treated group to better visualize changes resulting from treatment conditions. Blood sugar values in water-treated fish were also reduced compared with those in untreated fish directly removed from stock tanks. However, this has been reported previously (Connaughton et al., 2016) and reflects differences in the frequency of feeding and fasting prior to blood sugar measurements.

### Glucose treatment suppressed b-wave amplitudes owing to selective effects on R, G and B cones

ERG b2 amplitudes isolated from the distal retina by CNQX treatment were significantly decreased in glucose-treated tissue at all stimulus wavelengths. As only cones and ON bipolar cells respond to light in these conditions, these ERG deficits originate from cones and their synapses onto ON bipolar cells. Our results further suggest that R cone signals are robustly susceptible to hyperglycemic insult, regardless of adaptation state. Susceptibility of G and B cones was also revealed using red adaption, where these types are essentially dark adapted. These results concur with reported glucose-induced anatomical changes to zebrafish cones (Alvarez et al., 2010) and suggest that the anatomical changes are correlated with functional deficits.

Native ERG b-wave amplitudes from eyecups not perfused with CNQX were decreased in glucose-treated fish (Alvarez et al., 2010). These white-light responses reflect signals from all cone and neuronal types. ERG d-waves were previously reported to decrease in glucose-treated tissue (Alvarez et al., 2010), a finding we replicate. The current, more extensive, data provided in Table S1 and Fig. S3 show that a-wave amplitudes increased in response to glucose treatment, and a-, b- and d-wave implicit times became quicker.

A surprising finding was the large amplitude response of mannitol-treated tissue versus water controls. Previous studies (Alvarez et al., 2010) used untreated fish as a water control, and thus did not encounter this effect. In other studies by our group (Connaughton et al., 2016; Gleeson et al., 2007), in most parameters assessed here (blood sugar, wet weight, overall health) and in cell culture studies that use mannitol as an osmotic control for glucose treatment (Chen et al., 2013; Costa et al., 2013), the mannitol group reflects control (Chen et al., 2013; Gillies et al., 1997; Santiago et al., 2007) and/or normoglycemic (Dennis et al., 2015; Schrufer et al., 2010) responses. However, our ERG recordings revealed differences between control groups. The reasons for this are unknown but they could be due to either a hyperosmotic response or a protective effect of mannitol. Our mannitol and glucose treatment groups used the same concentrations (2%) and the same exposure regime. However, it is likely that glucose was metabolized by the fish as they have functioning insulin and insulin receptors. Not all ingested glucose is immediately stored, however, as evidenced by high blood sugar levels in the treatment phase (Table 1C). Mannitol is not taken up by cells nor does it affect blood sugar levels, suggesting hyperosmotic effects, potentially localized to the cellular level. Exposure to hyperosmotic solutions increases spontaneous quantal release of neurotransmitter at frog neuromuscular junctions (Kashani et al., 2001) and enhances transmitter release onto ganglion cells in retinal slices (Kashani et al., 2001). In mice, however, mannitol can be metabolized by gut bacteria (Xiao et al., 2013), indirectly providing nutritional benefit, not manifesting as deleterious high serum glucose.

Alternatively, mannitol could be protective to retinal tissue. Clinically, mannitol is used in the treatment of glaucoma (Jin et al., 2007; Lam et al., 2002; Nagaraju et al., 2007; Rhee et al., 2006), as an adjuvant to facilitate stem cell delivery to the brain (Gonzales-Portillo et al., 2014) and to reduce ischemic damage in cardiac muscle cells (Powell et al., 1976). Similar effects might be occurring in the retina. However, perfusion with a mannitol solution disrupts the blood-brain barrier in rodents (Kaya et al., 2004). Intravitreal injection of mannitol causes retinal detachment (Marmor, 1979; Marmor et al., 1980), alters retinal pigment epithelium (RPE) c-wave activity (Marmor, 1979; Shirao and Steinberg, 1987) and, over time, decreases ERG a- and b-wave components (Marmor, 1979). Whatever the physiological mechanism underlying mannitol's potential protective effect on retinal tissue, our data suggest that it is long-term, as there was no mannitol in the eyecup perfusate during recordings.

Altered ERG implicit times and response amplitudes (Klemp et al., 2004), visual-evoked potentials and pattern ERG responses (Parisi et al., 1998) can be observed prior to any clinical signs of retinopathy (Tzekov and Arden, 1999). These physiological changes increase in severity with DR progression (Tzekov and Arden, 1999; Zakareia et al., 2010). In rodents, changes in ERG a- and/or b-wave components do not begin immediately after hyperglycemic induction. Rather, most are reported to occur after 10-12 weeks (Bui et al., 2009; Hancock and Kraft, 2004; Li et al., 2002; Phipps et al., 2006; Ramsey et al., 2006), when retinal thinning is observed (Martin et al., 2004), although some reports indicate that changes can occur earlier, at ∼6 weeks (Layton et al., 2007; Li et al., 2002). In diabetic patients, disease progression occurs over many years. Thus, in both rodents and humans, there is a time course associated with hyperglycemia-induced changes in retinal function. This time course appears to be accelerated in the zebrafish model.

### Retinal anatomy

No changes in retinal thickness were observed in glucose- versus mannitol-treated tissue, similar to previously reported results in Epon-embedded sections (Alvarez et al., 2010), but different from measurements in cryostat sections (Gleeson et al., 2007). Glucose-induced differences in cone morphology have also been reported, with the greatest defects observed in double (R/G) cones (Alvarez et al., 2010). Our physiological data support photoreceptor-specific deficits, even though we did not detect any morphological differences in the photoreceptor layer in our thick sections.

GFAP and Nf-κB content increased in glucose-treated retinal homogenates, with a large, significant increase in Nf-κB. Early stages of DR are associated with glucose-induced upregulation of pro-inflammatory and pro-apoptotic markers (Adamis and Berman, 2008; Joussen et al., 2001; Kern, 2007), which stimulate leukostasis in capillary endothelial cells (Adamis and Berman, 2008; Kern, 2007; Tang and Kern, 2011), resulting in vessel leakage, capillary dropout and other changes characterizing the clinical onset of DR. Experiments have identified a common set of inflammatory markers, including Nf-κB, that are activated by high glucose (Kern, 2007; Romeo et al., 2002; Tang and Kern, 2011). The 3.5× increase in Nf-κB in glucose-treated retinas observed here is consistent with a hyperglycemia-induced inflammatory response. GFAP levels were also elevated in glucose-treated retinas, but not significantly. Previous reports indicate an increase in GFAP levels only in mannitol-treated tissue, suggesting an osmotic effect (Alvarez et al., 2010). Upregulation of GFAP occurs in response to hyperglycemia (Barber et al., 2000; Gerhardinger et al., 2005; Lieth et al., 1998; Mizutani et al., 1998) as well as other retinal insults, including light damage (Grosche et al., 1995), photoreceptor degeneration (DiLorento et al., 1995; Lewis et al., 1995) and retinal detachment (Lewis et al., 1995).

## Conclusions

Zebrafish exposed to 1 month of alternating 2% glucose/0% glucose display elevated blood sugar levels. Retinal homogenates identified upregulation of Nf-κB, suggesting an inflammatory response. Using spectrally distinct stimuli and AMPA/KA receptor blockade in the inner retina, glucose exposure decreased a1 and b2 response amplitudes, owing to an impairment primarily between R cones and their ON bipolar synapses. In response to bright-white light, native ERGs from glucose-treated eyecups displayed reduced b- and d-wave amplitudes and decreased implicit times for a-, b- and d-waves; effects on a-wave amplitude depended on stimulus brightness. These findings indicate that prolonged hyperglycemia adversely affects zebrafish retinal electrophysiology through separate actions in both distal and proximal retina, with distal effects on b-wave amplitudes and proximal effects on response component implicit times.

## MATERIALS AND METHODS

### Animals

Wild-type adult zebrafish (*Danio rerio*) (4 months to 1 year) of mixed sex were purchased from a vendor or bred from the in-house zebrafish colony and housed within the Fish Facility at American University. All procedures were approved by the American University Institutional Animal Care and Use Committee. The fish were acclimated for at least 1 week to standard laboratory conditions (28°C; 14 h light/10 h dark) prior to experiments. The fish were fed a diet of Tetramin flakes+zebrafish crumble (Aquatic Habitats) daily until the beginning of the experiments.

### Exposure protocol

Hyperglycemia was induced in zebrafish by exposing the fish to alternating conditions of 2% glucose/0% glucose every 24 h (i.e. a change in solution composition was made every 24 h) (Gleeson et al., 2007). Osmotic control animals were exposed to 2% mannitol/0% mannitol every 24 h and the water-treated control animals (for handling stress) were exposed to 0% glucose/0% glucose (water/water solution change) every 24 h. Deer Park Spring Water was used to make the experimental solutions. Fish were fed every other day while in the experimental containers. Temperature, pH, water quality and general fish behavior were recorded daily. Fish were maintained on the alternating exposure protocol for at least 28 days (4 weeks) before ERG analysis. Starting on day 28, 2 fish were removed and tested each day. Alternations between the 2 solutions continued for all remaining fish until they were analyzed for ERG responsiveness.

At the end of the 28-day exposure period, a subset of fish from each of the 3 treatment groups was removed from treatment and anesthetized in 0.02% tricaine solution. For some experiments, untreated fish taken directly from stock tanks were also used. Once anesthetized, the fish were weighed (wet weight, in mg; Sartorius microbalance) and decapitated so that a blood sugar measurement could be obtained (Connaughton et al., 2016; Gleeson et al., 2007). Blood sugar was measured from cardiac blood using a Freestyle-Lite Blood Glucose Meter (Connaughton et al., 2016). Differences in wet weight and blood sugar were assessed for outliers using Dixon's outlier test and then compared across treatments using a one-way analysis of variance (SPSS software) at an α-level of 0.05.

### ERG recordings

Fish were sacrificed by decapitation. Heads were hemisected to remove the eyes and the anterior segment was removed, forming an eyecup. Each eyecup was placed in the recording chamber and superfused with an oxygenated (95% O_2_/5% CO_2_, 20°C) minimal essential medium (MEM) solution (Millipore) for at least 30 min. A 28-gauge nonmetalic syringe needle (WPI, Sarasota, FL, USA) was placed inside the eyecup to perfuse the retina at a rate of 0.3 ml min^−1^. For responses using spectrally distinct stimuli, the MEM contained 50 µM CNQX, an AMPA/KA receptor antagonist (Tocris), to isolate a- and b-wave responses. Under these conditions, the major b-wave component is the b2 response (Nelson and Singla, 2009), which is the non-AMPA/KA receptor response of the ON bipolar cells postsynaptic to stimulated cone photoreceptors. The major a-wave component is the a1 response (Nelson and Singla, 2009). For ERG responses elicited by white-light stimuli, CNQX was not used, allowing native a-wave, b-wave and d-wave components to be recorded. Glucose, mannitol and water treatments were not continued during the recording sessions, although all recordings were made in 0.1% glucose, the amount standard to MEM.

These photopic ERG signals were amplified (DAM80, World Precision Instruments) using a bandpass from 0.1 Hz to 1 kHz and a Digidata 1440A (Axon Instruments) with a sampling rate of 2 kHz. Traces averaged 4× for each stimulus condition were boxcar filtered over a period corresponding to one cycle of 60 Hz line frequency. Mean response component amplitudes, peak times and spectral properties were obtained from the different treatment groups. Data were collected with pCLAMP 10 software (Axon Instruments) and analyzed using Origin 8.5, 9.0 or 9.1 (2015, 2016).

### White-light stimuli

A white-light stimulus was tested on 10 eyes per treatment group. White-light ERG responses had a-wave, b-wave and d-wave components (Fig. 1A). In the white-light protocol, the background was infrared (IR, RG780 filter). The white-light protocol generated a response dataset with 70, 4-trace-average ERGs. These ERGs were in response to 10 replicates of a 7-step irradiance response series covering a 3-log unit range. The white-light source was a 150 W Xenon arc lamp, as imaged through UV compliant optics, providing a flat energy spectrum across the visual sensitivity range of zebrafish from 650 nm to 370 nm. This range includes zebrafish UV cones, as well as R, G and B cones. The maximum delivered irradiance (−3.0 log units attenuation) was 0.15 µW cm^−2^. The white-light protocol was run 3× on each eyecup, covering a recording period of ∼75 min, to generate a total of 30 ERGs for each brightness level from each eye, or a total 300 ERGs at each of 7 brightness levels from 10 eyes.

For statistical comparisons between treatments, all white-light datasets were combined into a single, cumulative dataset for each treatment group. This data analysis was consistent with the modeling analysis of spectral responses (see below). Amplitudes of a-, b- and d-waves were measured as means within fixed, characteristic, time intervals after onset or cessation of the stimulus (pre-stimulus baselines set to ‘0’). The time intervals were selected to include a-, b- or d-wave peaks. This method allowed consistent measurements of response amplitude as the time-to-peak for each component varied with treatment. The native ERG a-wave amplitude (μV) was the mean amplitude within 15-35 ms after stimulus onset. The a-wave trough time (ms) was the time of maximal excursion during a 10-100 ms poststimulus interval. Both b-wave amplitude (μV) and peak times (ms) were measured in the interval 51-200 ms after stimulus onset. For the d-wave, amplitude and peak times were measured in the interval 25-250 ms after stimulus offset. Means during characteristic time segments were always used as amplitude indexes for a, b or d-waves, but in the absence of a peak within an interval, no implicit time was scored.

### Spectral stimuli

In a separate set of experiments, 7-15 eyes per treatment were tested with spectral stimuli. The spectral protocol used 9 wavelengths ranging from 330 nm to 650 nm (40 nm increments, 20 nm half bandwidth) over 7 intensities (0.5 log unit increments covering 3 log units). The spectral protocol generated a response dataset with 70, 4-trace-average ERGs. These ERGs were in response to 64 unique combinations of wavelength and intensity, with the remaining 6 ERGs being replicates. Mean amplitudes and peak times of ERG components were measured as described above, except the a1-wave amplitude time segment was 10-65 ms, because the a1-wave, as revealed in CNQX, is slower than the native a-wave.

Two chromatic backgrounds were used to better isolate individual cone mechanisms and to prevent rod dark adaptation: red (627 nm) and blue (418 nm). The former background helped to isolate G, B and UV cone signals from the R cone signal, while the latter reduced the B cone signal and, to a lesser extent, the G cone signal in comparison to the R and UV cone signals. The irradiance of the red background was 4.79 log(quanta µm^−2^ s^−1^) and the irradiance of the blue background was 3.84 log(quanta µm^−2^ s^−1^). The spectral protocol was run on each eyecup 4 times, twice with each background. Recordings were accepted if amplitudes and waveforms remained stable during the 17 min, 70-stimulus protocol, as stability is critical to spectral analysis. The resulting ERG recordings had characteristic a (a1)- and b (b2)-waves (Fig. 1B).

### Spectral properties of treated groups

The Nelson and Singla (2009) spectral analysis protocol for zebrafish ERGs was used to evaluate separately the extent of the R, G, B and UV cone signals contributing to the amplitude of the ERG waveform and to the overall spectral performance of the eye. Here, we use this analysis to evaluate individual cone contributions to b2 amplitudes in spectral datasets from the CNQX treatment groups. Spectral responses were modeled as a linear sum of 4 Hill functions (Eqn 1). Each Hill function represented the intensity response characteristic for signals from one of the cone types:(1)
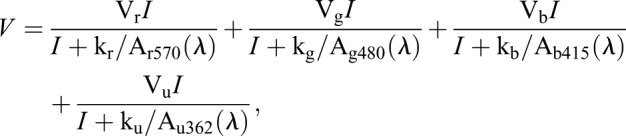
where *V* is the b2-wave response amplitude, *I* is the stimulus irradiance and *λ* is the stimulus wavelength; V_r_, V_g_, V_b_, and V_u_ are the saturated amplitudes of the R, G, B, and UV cone signals, respectively; k_r_, k_g_, k_b_ and k_u_ are the half-saturation irradiances for these signals at the opsin absorbance maxima; and A_r570_, A_g480_, A_b415_ and A_u362_ are the polynomial absorbance spectra of the R, G, B and UV pigments normalized to peak sensitivity. (These opsin spectral functions are graphed in Fig. 1C; comparison of stimulus discrimination is provided in Table S1.) Overall, 151 spectral datasets fulfilled the stability recording criteria and were accepted for analysis. The median r^2^ for nonlinear least squares fits of individual datasets to the model (Eqn 1) was 0.98 (Origin). In the spectral analysis, b2 amplitudes were measured more conventionally, from a1-wave trough to b2-wave peak (Samuels et al., 2012; Tzekov and Arden, 1999).

Treatment-level datasets included all the responses from all eyes in each treatment group, allowing the model to run properly and to provide a detailed analysis of changes in cone inputs across the different stimulus intensities and wavelengths. The model also reports the number of responses used for each analysis; we provide this value as the N in the results. We chose to use the spectral model to examine hyperglycemia-induced effects on cone photoreceptors when analysis using average responses across eyes revealed no significant differences across treatment, owing to the higher variability, and lability, in the responses from eyes of glucose-treated fish (Fig. S3). However, differences in ERG traces due to treatment are clearly apparent (Figs 2-4). By using all the data points in each dataset, the spectral model identified and quantified these differences. This spectral model has been used previously to quantify the magnitude of inputs from the different cone photoreceptors in zebrafish horizontal (Connaughton and Nelson, 2010) and amacrine (Torvund et al., 2017) cells.

### Statistical analysis of ERG data

Our experiments utilized two control groups: an osmotic control (mannitol) and a handling stress/transfer control (water). The mannitol treatment controls for the increased osmolarity due to the presence of glucose in tank water and the water treatment controlled for daily transferring of the fish. Interestingly, in all ERG recordings, the response amplitudes from the water-treated control group were consistently the lowest recorded. We believe that this reflects an effect of handling stress/daily transfers, even though blood sugar levels were not elevated in these fish (Table 1A) and they displayed no overt stressful behavior(s). Responses from the water-treated control group were also reduced compared with responses from untreated fish directly removed from stock tanks (Fig. 2A), again suggesting handling stress. Given that fish in the glucose and mannitol treatment groups were also transferred daily and would, therefore, experience similar handling stress(es), we normalized the ERG response amplitudes of the glucose- and mannitol-treated fish to those of the water treatment group to more accurately index the effects of hyperglycemia.

Statistical analyses were performed using SPSS ver. 22 software (IBM Corporation), Prism ver 6 (GraphPad Software) or Origin (OriginLab) and evaluated at α=0.05; graphs were generated in SigmaPlot (ver. 12) or Origin. Statistical differences within each parameter at each wavelength were assessed using either a one-way analysis of variance (ANOVA) or a Student's *t*-test with α=0.05. If the results of the ANOVA were significant, a Tukey post hoc multiple comparison test was used.

### Anatomical analysis

To determine whether there were any changes in retinal layer morphology due to glucose exposure, 5 fish per treatment group were euthanized in 0.02% tricaine solution. Fish were then decapitated, and the eyes were removed and fixed in 4% paraformaldehyde. Each eye was washed 3× in 0.9% saline solution and dehydrated through an ethanol series (30%, 50%, 70%, 95%, 100%). Tissue was then washed 2× with either propylene oxide or acetone and infiltrated in a 1:1 propylene oxide: resin or acetone: resin solution for 1.5-2 h, followed by overnight infiltration in 1:2 solutions (propylene oxide: resin). The next day, tissue was embedded in fresh resin and allowed to polymerize for 3 days at 60°C.

Thick (3-5 µm) sections were cut with a diamond knife on a Leica UL-7 ultramicrotome. Sections were mounted on gelatin-coated glass slides and stained with a mixture of 1% Methylene Blue, 1% Azure A, and 1% sodium borate (Richardson et al., 1960; Tarboush et al., 2014). Once dried, sections were viewed at 5×, 10× and 40× using a Zeiss inverted microscope, photographed using QCapture Plus software (ver. 3.1.3.10) and measured using ImageJ (National Institutes of Health).

Measurements of retinal layer thickness were made from sections of central retina, behind the lens. Thickness of the whole retina, IPL, INL, OPL, ONL and photoreceptor layer (PL; from the distal side of the OPL to the outer edge of the retinal pigment epithelium) were measured in the vitread-sclerad direction. Each measurement was made 3× and then averaged to reduce error. For consistency, only sections with an identified cornea and lens were measured. A one-way ANOVA was used to determine significant differences between mean thickness measurements across treatment, followed by a Tukey post hoc test (α=0.05).

### Western blots

Following measurement of blood sugar, eyes were enucleated, and the cornea and lens removed. Retinal tissue was then separated from the sclera and placed into RIPA buffer (3-5 retinas per replicate per treatment) on ice. Total protein lysates were prepared from pooled retinas using M-PER reagent (Thermo Fisher Scientific, Rockford, IL, USA) containing Halt protease/phosphatase inhibitor (Thermo Fisher Scientific) in accordance with the manufacturer's protocol. Proteins were quantified using a BCA protein assay kit (Thermo Fisher Scientific, Waltham, MA, USA) and separated using 4-12% Bis-Tris protein gels (Life Technologies, Grand Island, NY, USA). Gels were electrophoresed and transferred to polyvinylidene difluoride membrane using the iBlot 2 Dry Blotting System (Life Technologies). Membranes were blocked in 5% bovine serum albumin (BSA). Primary GFAP, RelA (V-Rel Avian Reticulodneotheliosis Viral Oncogene Homolog A) and β-actin antibodies were applied in a 1:1000 dilution in 5% BSA. Anti-rabbit (RelA, β-actin) or anti-mouse (GFAP) horseradish peroxidase-conjugated secondary antibodies were applied at a dilution of 1:2000 (Cell Signaling Technology, Danvers, MA, USA). West Dura Chemiluminescent substrate (Thermo Fisher Scientific) was used for signal detection. Membranes were visualized using a ChemiDoc-It imaging system (UVP, Upland, CA, USA) and densitometry was quantified using ImageJ. Results were normalized to β-actin, which served as a loading control.

## Supplementary Material

Supplementary information

## References

[DMM035220C1] Acerete L., Balasch J. C., Espinosa E., Josa A. and Tort L. (2004). Physiological responses in Eurasian perch (Perca fluviatilis, L.) subjected to stress by transport and handling. *Aquaculture* 237, 167-178. 10.1016/j.aquaculture.2004.03.018

[DMM035220C2] Adamis A. P. and Berman A. J. (2008). Immunological mechanisms in the pathogenesis of diabetic retinopathy. *Semin. Immunopathol.* 30, 65-84. 10.1007/s00281-008-0111-x 18340447

[DMM035220C3] Agardh E., Bruun A. and Agardh C.-D. (2001). Retinal glial cell immunoreactivity and neuronal cell change sin rats with STZ-induced diabetes. *Curr. Eye Res.* 23, 276-284. 10.1076/ceyr.23.4.276.5459 11852429

[DMM035220C4] Alvarez Y., Cederlund M. L., Cottell D. C., Bill B. R., Ekker S. C., Torres-Vazquez J., Weinstein B. M., Hyde D. R., Vihtelic T. S. and Kennedy B. N. (2007). Genetic determinants of hyaloid and retinal vasculature in zebrafish. *BMC Dev. Biol.* 7, 114-130. 10.1186/1471-213X-7-114 17937808 PMC2169232

[DMM035220C5] Alvarez Y., Chen K., Reynolds A., Waghorne N., O'Connor J. and Kennedy B. (2010). Predominant cone photoreceptor dysfunction in a hyperglycaemic model of non-proliferative diabetic retinopathy. *Dis. Model. Mech.* 3, 236-245. 10.1242/dmm.003772 20142328

[DMM035220C6] Arden G. B. and Ramsey D. J. (2015). Diabetic retinopathy and a novel treatment based on the biophysics of rod photoreceptors and dark adaptation. In *Webvision: the Organization of the Retina and Visual System [internet]* (ed. Kolb H., Fernandez E. and Nelson R.). University of Utah Health Sciences Center. 26247094

[DMM035220C7] Argenton F., Zeccin E. and Bortolussi M. (1999). Early appearance of pancreatic hormone-expressing cells in the zebrafish embryo. *Mech. Dev.* 87, 217-221. 10.1016/S0925-4773(99)00151-3 10495291

[DMM035220C8] Barber A. J., Antonetti D. A., Gardner T. W. and Group T. P. S. R. R. (2000). Altered expression of retinal occludin and glial fibrillary acidic protein in experimental diabetes. *Invest. Ophthalmol. Vis. Sci.* 41, 3561-3568. 11006253

[DMM035220C9] Bui B. V., Loeliger M., Thomas M., Vingrys A. J., Rees S. M., Nguyen C. T. O., He Z. and Tolcos M. (2009). Investigating structural and biochemical correlates of ganglion cell dysfunction in streptozotocin-induced diabetic rats. *Exp. Eye Res.* 88, 1076-1083. 10.1016/j.exer.2009.01.009 19450451

[DMM035220C10] Capiotti K., Junior R., Kist L., Bogo M., Bonan C. and da silva R. (2014). Persistent impaired glucose metabolism in zebrafish hyperglycemia model. *Comp. Biochem. Physiol. B* 171, 58-65. 10.1016/j.cbpb.2014.03.005 24704522

[DMM035220C11] Carrasco E., Hernandez C., de Torres I., Farres J. and Simo R. (2008). Lowered cortistatin expression is an early event in the human diabetic retina and is associated with apoptosis and glial activation. *Mol. Vis.* 14, 1496-1502. 18709137 PMC2516506

[DMM035220C12] Castilho A., Ambrosio A., Hartveit E. and Veruki M. (2015). Disruption of neural microcircuitry in the rod pathway of the mammalia retina by diabetes mellitus. *J. Neurosci.* 35, 5422-5433. 10.1523/JNEUROSCI.5285-14.2015 25834065 PMC6705407

[DMM035220C13] Ceriello A., Esposito K., Piconi L., Ihnat M., Thorpe J., Testa R., Boemi M. and Giugliano D. (2008). Oscillating glucose is more deleterious to endothelial cell function and oxidative stress than mean glucose in normal and type 2 diabetic patients. *Diabetes* 57, 1349-1354. 10.2337/db08-0063 18299315

[DMM035220C14] Chapman G. B., Tarboush R., Eagles D. A. and Connaughton V. P. (2009). A light and transmission electron microscope study of the distribution and ultrastructural features of peripheral nerve processes in the extra-retinal layers of the zebrafish eye. *Tissue Cell* 41, 286-298. 10.1016/j.tice.2008.12.003 19251294

[DMM035220C15] Chen X.-L., Zhang X.-D., Li Y.-Y., X-M C., Tang D.-R. and Ran R.-J. (2013). Involvement of HMGB1 mediated signalling pathway in diabetic retinopathy: evidence from type 2 diabetic rats and ARPE-19 cells under diabetic condition. *J. Ophthalmol.* 97, 1598-1603. 10.1136/bjophthalmol-2013-303736 24133029

[DMM035220C16] Connaughton V. P. and Nelson R. (2010). Spectral responses in zebrafish horizontal cells include a tetraphasic response and a novel UV-dominated triphasic response. *J. Neurophysiol.* 104, 2407-2422. 10.1152/jn.00644.2009 20610786 PMC2997023

[DMM035220C17] Connaughton V. P., Baker C., Fonde L., Gerardi E. and Slack C. (2016). Alternate immersion in an external glucose solution differentially affects blood sugar values in older vs. *youner zebrafish adults*. *Zebrafish* 13, 87-94. 10.1089/zeb.2015.1155 26771444

[DMM035220C18] Costa E., Barros N., Coppin L., Neves R., Carmona A., Penha F., Rodrigues E. D., Dib E., Magalhães O. J., Moraes-Filho M. et al. (2013). Effects of light exposure, pH, osmolarity, and solvent on the retinal pigment epithelial toxicity of vital dyes. *Am. J. Ophthalmol.* 155, 705-712. 10.1016/j.ajo.2012.10.004 23253911

[DMM035220C19] De Lombaert M., Rick E., KKrugner-Higby L. and Walman M. (2017). Behavioral characteristics of adult zebrafish (Danio rerio) after MS222 anesthesia for fin excision. *J. Am. Assoc. Lab. Anim. Sci.* 56, 377-381. 28724486 PMC5517326

[DMM035220C20] Dennis M. D., Kimball S. R., Fort P. E. and Jefferson L. S. (2015). Regulated in development and DNA damage 1 is necessary for hyperglycemia-induced vascular endothelian growth factor expression in the retina of diabetic rodents. *J. Biol. Chem.* 290, 3865-3874. 10.1074/jbc.M114.623058 25548280 PMC4319049

[DMM035220C21] DiLorento D. Jr, Martzen M., del Cerro C., Coleman P. and del Cerro M. (1995). Muller cell changes precede photoreceptor cell degeneration in the age-related retinal degeneration of the Fischer 344 rat. *Brain Res.* 698, 1-14. 10.1016/0006-8993(95)00647-9 8581466

[DMM035220C22] Enrico P., Bouma M., de Vries J. B. and Westerink B. H. C. (1998). The role of afferents to the ventral tegmental area in the handling stress-induced increae in the release of dopamine in the medial prefrontal cortex: a dual-probe microdialysis study in the rat brain. *Brain Res.* 779, 205-213. 10.1016/S0006-8993(97)01132-3 9473673

[DMM035220C23] Gerhardinger C., Costa M. B., Coulombe M. C., Toth I., Hoehn T. and Grosu P. (2005). Expression of acute-phase response proteins in retinal Muller cells in diabetes. *Invest. Ophthalmol. Vis. Sci.* 46, 349-357. 10.1167/iovs.04-0860 15623795

[DMM035220C24] Gillies M., Su T., Stayt J., Simpson J., Naidoo D. and Salonikas C. (1997). Effect of high glucose on permeability of retinal capillary endothelium in vitro. *Invest. Ophthalmol. Vis. Sci.* 38, 635-642. 9071217

[DMM035220C25] Gleeson M., Connaughton V. P. and Arneson L. (2007). Induction of hyperglycaemia in zebrafish (*Danio rerio*) leads to morphological changes in the retina. *Acta Diabetol.* 44, 157-163. 10.1007/s00592-007-0257-3 17721755

[DMM035220C26] Gonzales-Portillo G., Sanberg P., Franzglau M., Gonzales-Portillo C., Diamandis T., Staples M., Sanberg C. and Borlongan C. (2014). Mannitol-enhanced delivery of stem cells and their growth factors across the blood-brain barrier. *Cell Transplant.* 23, 531-539. 10.3727/096368914X678337 24480552 PMC4083632

[DMM035220C27] Grosche J., Härtig W. and Reichenbach A. (1995). Expression of glial fibrillary acidic protein (GFAP), glutamine synthetase (GS), and Bcl-2 protooncogene protein by Muller (glial) cells in retinal light damage in rats. *Neurosci. Lett.* 185, 119-122. 10.1016/0304-3940(94)11239-F 7746501

[DMM035220C28] Guthrie R. A. and Guthrie D. W. (2004). Pathophysiology of diabetes mellitus. *Crit. Care Nurs. Q* 27, 113-125. 10.1097/00002727-200404000-00003 15137354

[DMM035220C29] Hancock H. A. and Kraft T. W. (2004). Oscillatory potential analysis and ERGs of normal and diabetic rats. *Invest. Ophthalmol. Vis. Sci.* 45, 1002-1008. 10.1167/iovs.03-1080 14985323

[DMM035220C30] Hughes A., Saszik S., Bilotta J., Demarco P. Jr. and Patterson W. N. (1998). Cone contributions to the photopic spectral sensitivity of the zebrafish ERG. *Visual Neurosci.* 15, 1029-1037. 10.1017/S095252389815602X 9839967

[DMM035220C31] Intine R., Olsen A. and Sarras M. (2013). A zebrafish model of diabetes mellitus and metabolic memory. *J. Vis. Exp.* 72, e50232 10.3791/50232 PMC3622110 23485929

[DMM035220C32] Jin X., Xu A., Zhao Y., Qin Q., Dong X.-D. E. and Qu J. (2007). Efficacy and safety of intravenous injection of lidocaine in the treatment of acute primary angle-closure glaucoma: a pilot study. *Graeffes Arch. Clin. Exp. Ophthalmol.* 245, 1611-1616. 10.1007/s00417-007-0572-y 17437125

[DMM035220C33] Joussen A., Huang S., Poulaki V., Camphausen K., Beecken W.-D., Kirchhof B. and Adamis A. (2001). In vivo retinal gene expression in early diabets. *Invest. Ophthalmol. Vis. Sci.* 42, 3047-3057. 11687554

[DMM035220C34] Kashani A., Chen B.-M. and Grinnell A. (2001). Hypertonic enhancement of transmitter release from frog motor nerve terminals: Ca+2 independence and role of integrins. *J. Physiol.* 530, 243-252. 10.1111/j.1469-7793.2001.0243l.x 11208972 PMC2278411

[DMM035220C35] Kaya M., Gulturk S., Elmas I., Kalayci R., Arican N., Kocyildiz Z., Kucuk M., Yorulmaz H. and Sivas A. (2004). The effects of magnesium sulfact on blood-brain barrier disruption caused by intracarotid injection of hyperosmolar mannitol in rats. *Life Sci.* 76, 201-212. 10.1016/j.lfs.2004.07.012 15519365

[DMM035220C36] Kern T. S. (2007). Contributions of inflammatory processes to the development of the early stages of diabetic retinopathy. *Exp. Diabetes Res.* 2007, 95103 10.1155/2007/95103 18274606 PMC2216058

[DMM035220C37] Kinkel M. D. and Prince V. E. (2009). On the diabetic menu: zebrafish as a model for pancreas development and function. *BioEssays* 31, 139-152. 10.1002/bies.200800123 19204986 PMC2770330

[DMM035220C38] Kizawa J., Machida S., Kobayashi T., Gotoh Y. and Kurosaka D. (2006). Changes in oscillatory potentials and photopic negative response in patients with early diabetic retinopathy. *Jpn. J. Ophthalmol.* 50, 367-373. 10.1007/s10384-006-0326-0 16897223

[DMM035220C39] Klemp K., Larsen M., Sander B., Vaag A., Brockhoff P. B. and Lund-Andersen H. (2004). Effect of short-term hyperglycemia on multifocal electroretinogram in diabetic patients without retinopathy. *Invest. Ophthalmol. Vis. Sci.* 45, 3812-3819. 10.1167/iovs.03-1260 15452093

[DMM035220C40] Kohzaki K., Vingrys A. J. and Bui B. V. (2008). Early inner retinal dysfunction in streptozotocin-induced diabetic rats. *Invest. Ophthalmol. Vis. Sci.* 49, 3595-3604. 10.1167/iovs.08-1679 18421077

[DMM035220C41] Kowluru R. A. and Chan P.-S. (2007). Oxidative stress and diabetic retinopathy. *Exp. Diabetes Res.* 2007, 43603 10.1155/2007/43603 17641741 PMC1880867

[DMM035220C42] Lam D. S. C., Chua J. K. H., Tham C. C. Y. and Lai J. S. M. (2002). Efficacy and safety of immediate anterior chamber paracentesis in the treatment of acute primary angle-closure glaucoma. *Ophthalmol* 109, 64-70. 10.1016/S0161-6420(01)00857-0 11772581

[DMM035220C43] Layton C. J., Safa R. and Osborne N. N. (2007). Oscillatory potentials and the b-wave: partial masking and interdependence in dark adaptation and diabetes in the rat. *Graefe's Arch. Clin. Exp. Ophthalmol.* 245, 1335-1345. 10.1007/s00417-006-0506-0 17265029

[DMM035220C44] Lee J., Jung D.-W., Kim W.-H., Um J.-I., Yim S.-H., Oh W. K. and Williams D. R. (2013). Development of a highly visual, simple, and rapid test for the discovery of novel insulin mimetics in living vertebrates. *ACS Chem. Biol.* 8, 1803-1814. 10.1021/cb4000162 23725454

[DMM035220C45] Lewis G., Matsumoto B. and Fisher S. (1995). Changes in the organization and expression of cytoskeletal proteins during retinal degeneration induced by retinal detachment. *Invest. Ophthalmol. Vis. Sci.* 36, 2404-2416. 7591630

[DMM035220C46] Li Q., Zemel E., Miller B. and Perlman I. (2002). Early retinal damage in experimental diabetes: electroretinographical and morphological observations. *Exp. Eye Res.* 74, 615-625. 10.1006/exer.2002.1170 12076083

[DMM035220C47] Lieth E., Barber A. J., Xu B., Dice C., Ratz M. J., Tanase D., Strother J. M. and Group T. P. S. R. R. (1998). Glial reactivity and impaired glutamate metabolism in short-term experimental diabetic retinopathy. *Diabetes* 47, 815-820. 10.2337/diabetes.47.5.815 9588455

[DMM035220C48] Lung J. C. Y., Swann P. G. and Chan H. H. L. (2016). The multifocal on- and off-responses in the human diabetic retina. *PLoS ONE* 11, e0155071 10.1371/journal.pone.0155071 27187490 PMC4871365

[DMM035220C49] Marmor M. (1979). Retinal detachment from hyperosmotic intravitreal injection. *Invest. Opthal. Vis. Sci.* 18, 1237-1244. 116971

[DMM035220C50] Marmor M., Martin L. and Tharpe S. (1980). Osmotically induced retinal detachment in the rabbit and primate. *Invest. Ophthalmol. Vis. Sci.* 19, 1016-1029. 7409995

[DMM035220C51] Martin P. M., Roon P., Van Ells T. K., Ganapathy V. and Smith S. B. (2004). Death of retinal neurons in streptozotocin-induced diabetic mice. *Invest. Ophthalmol. Vis. Sci.* 45, 3330-3336. 10.1167/iovs.04-0247 15326158

[DMM035220C52] Mizutani M., Gerhardinger C. and Lorenzi M. (1998). Muller cell changes in human diabetic retinopathy. *Diabetes* 47, 445-449. 10.2337/diabetes.47.3.445 9519752

[DMM035220C53] Nagaraju M., Saleh M. and Porciatti V. (2007). IOP-dependent retinal ganglion cell dysfunction in glaucomatous DBA/2J mice. *Invest. Opthalmol. Vis. Sci.* 48, 4573-4579. 10.1167/iovs.07-0582 PMC2031015 17898280

[DMM035220C55] Nelson R. and Singla N. (2009). A spectral model for signal elements isolated from zebrafish photopic electroretinogram. *Vis. Neurosci.* 26, 349-363. 10.1017/S0952523809990113 19723365 PMC2758326

[DMM035220C56] Olsen A. S., Sarras M. P. and Intine R. V. (2010). Limb regeneration is impaired in an adult zebrafish model of diabetes mellitus. *Wound Rep. Reg.* 18, 532-542. 10.1111/j.1524-475X.2010.00613.x PMC2941236 20840523

[DMM035220C57] Palacios A. G., Goldsmith T. H. and Bernard G. D. (1996). Sensitivity of cones from a cyprinid fish (Danio aequipinnatus) to ultraviolet and visible light. *Visual Neurosci.* 13, 411-421. 10.1017/S0952523800008099 8782369

[DMM035220C58] Parisi V., Uccioli L., Parisi L., Colacino G., Manni G., Menzinger G. and Bucci M. G. (1998). Neural conduction in visual pathways in newly-diagnosed IDDM patients. *Electroencephalogr. Clin. Neurophysiol.* 108, 490-496. 10.1016/S0168-5597(98)00026-4 9780019

[DMM035220C59] Patel S. and Santani D. (2009). Role of NF-KB in the pathogenesis of diabetes and its associated complications. *Pharmacol Reports* 61, 595-603. 10.1016/S1734-1140(09)70111-2 19815941

[DMM035220C60] Phipps J. A., Yee P., Fletcher E. L. and Vingrys A. J. (2006). Rod photoreceptor dysfunction in diabetes: activation, deactivation, and dark adaptation. *Invest. Ophthalmol. Vis. Sci.* 47, 3187-3194. 10.1167/iovs.05-1493 16799066

[DMM035220C61] Powell W. J., DiBona D. R., Flores J. and Leaf A. (1976). The protective effect of hyperosmotic mannitol in myocardial ischemia and necrosis. *Circulation* 54, 603-615. 10.1161/01.CIR.54.4.603 963849

[DMM035220C62] Ramsey D. J., Ripps H. and Qian H. (2006). An electrophysiological study of retinal function in the diabetic female rat. *Invest. Ophthalmol. Vis. Sci.* 47, 5116-5124. 10.1167/iovs.06-0364 17065533

[DMM035220C63] Rhee D. J., Ramos-Esteban J. C. and Nipper K. S. (2006). Rapid resolution of topiramate-induced angle-closure glaucoma with methylprednisolone and mannitol. *Am. J. Ophthalmol.* 141, 1133-1134. 10.1016/j.ajo.2006.01.021 16765687

[DMM035220C64] Richardson K. C., Jarett L. and Finke E. H. (1960). Embedding in epoxy resin for ultrathin sectioning in electron microscopy. *Stain. Technol.* 35, 313-323. 10.3109/10520296009114754 13741297

[DMM035220C65] Robinson J., Schmitt E. A., Harosi F. I., Reece R. I. and Dowling J. E. (1993). Zebrafish ultraviolet visual pigment: absorption spectrum, sequence, and localization. *Proc. Natl. Acad. Sci. USA* 90, 6009-6012. 10.1073/pnas.90.13.6009 8327475 PMC46856

[DMM035220C66] Romeo G., Liu W.-H., Asnaghi V., Kern T. S. and Lorenzi M. (2002). Activation of nuclear factor-KB induced by diabetes and high glucose regulates a proapoptotic program in retinal pericytes. *Diabetes* 51, 2241-2248. 10.2337/diabetes.51.7.2241 12086956

[DMM035220C67] Rubenstein A. L. (2003). Zebrafish: from disease modeling to drug discovery. *Curr. Opin. Drug Discov. Devel.* 6, 218-223. 12669457

[DMM035220C68] Rungger-Brandle E., Dosso A. A. and Leuenberger P. M. (2000). Glial reactivity, an early feature of diabetic retinopathy. *Invest. Ophthalmol. Vis. Sci.* 41, 1971-1980. 10845624

[DMM035220C69] Samuels I., Lee C.-A., Petrash J., Peachey N. and Kern T. (2012). Exclusion of aldose reductase as a mediator of ERG deficits in a mouse model of diabetic eye disease. *Visual Neurosci.* 29, 267-273. 10.1017/S0952523812000326 PMC3745719 23101909

[DMM035220C70] Samuels I. S., Bell B. A., Pereira A., Saxon J. and Peachey N. S. (2015). Early retinal pigment epithelium dysfunction is concomitant with hyperglycemia in mouse models of type 1 and type 2 diabetes. *J. Neurophysiol.* 113, 1085-1099. 10.1152/jn.00761.2014 25429122 PMC7132312

[DMM035220C71] Santiago A. R., Cristóvão A. J., Santos P. F., Carvalho C. M. and Ambrósio A. F. (2007). High glucose induces caspase-independent cell death in retinal neural cells. *Neurobiol. Dis.* 25, 464-472. 10.1016/j.nbd.2006.10.023 17239603

[DMM035220C72] Schrufer T. L., Antonetti D. A., Sonenberg N., Kimball S. R., Gardner T. W. and Jefferson L. S. (2010). Ablation of 4E-BP1/2 prevents hyperglycemia-mediated induction of VEGF expressionin the rodent retina and in Muller cells in culture. *Diabets* 59, 2107-2116. 10.2337/db10-0148 PMC2927931 20547975

[DMM035220C73] Shirao Y. and Steinberg R. (1987). Mechanisms of effects of small hyperosmotic gradients on the chick RPE. *Invest. Opthal. Vis. Sci.* 28, 2015-2025. 3679749

[DMM035220C74] Sieving P. (1993). Photopic ON- and OFF-pathway abnormalities in retinal dystrophies. *Trans. Am. Ophthalmol. Soc.* 91, 701-773. 8140708 PMC1298484

[DMM035220C75] Tang J. and Kern T. S. (2011). Inflammation in diabetic retinopathy. *Prog. Retin. Eye Res.* 30, 343-358. 10.1016/j.preteyeres.2011.05.002 21635964 PMC3433044

[DMM035220C76] Tarboush R., Novales Flamarique I., Chapman G. B. and Connaughton V. P. (2014). Variability in mitochondria of zebrafish photoreceptor ellipsoids. *Visual Neurosci.* 31, 11-23. 10.1017/S095252381300059X 24801620

[DMM035220C77] Timmerman W., Cisci G., Nap A., de Vries J. B. and Westerink B. H. C. (1999). Effects of handling on extracellular levels of glutamate and other amino acids in various areas of the brain measured by microdialysis. *Brain Res.* 833, 150-160. 10.1016/S0006-8993(99)01538-3 10375690

[DMM035220C78] Torvund M. M., Ma T. S., Connaughton V. P., Ono F. and Nelson R. F. (2017). Cone signals in monostratified and bistratified amacrine cells of adult zebrafish retina. *J. Comp. Neurol.* 525, 1532-1557. 10.1002/cne.24107 27570913 PMC6088789

[DMM035220C79] Tzekov R. and Arden G. B. (1999). The electroretinogram in diabetic retinopathy. *Surv. Ophthalmol.* 44, 53-60. 10.1016/S0039-6257(99)00063-6 10466588

[DMM035220C80] Xiao J., Li X., Min X. and Sakaguchi E. (2013). Mannitol improves absorption and retention of calcium and magnesium in growing rats. *Nutrition* 29, 325-331. 10.1016/j.nut.2012.06.010 23237654

[DMM035220C81] Xu J., Hu G., Huang T., Huang H. and Chen B. (2006). Using multifocal ERG responses to discriminate diabetic retinopathy. *Doc. Ophthalmol.* 112, 201-207. 10.1007/s10633-006-0006-x 16779498

[DMM035220C82] Zakareia F. A., Alderees A. A., Al Regaiy K. A. and Alrouq F. A. (2010). Correlation of electroretinography b-wave absolute latency, plasma levels of human basic fibroblast growth factor, vascular endothelial growth factor, soluble fatty acid synthase, and adrenomedullin in diabetic retinopathy. *J. Diabetes Complications* 24, 179-185. 10.1016/j.jdiacomp.2008.12.007 19216096

[DMM035220C83] Zeng X.-X., Ng Y.-K. and Ling E.-A. (2000). Neuronal and microglial response in the retina of streptozotocin-induced diabetic rats. *Visual Neurosci.* 17, 463-471. 10.1017/S0952523800173122 10910112

