# Supplementary material for: One month of hyperglycemia alters spectral responses of the zebrafish photopic electroretinogram
Source: Dis Model Mech. 2018 Oct 22;11(10):dmm035220. doi: 10.1242/dmm.035220 (PMC6215424; doi:10.1242/dmm.035220)

Supplementary Materials

Table S1. Mean pigment absorbance within the 20 nm half-bandwidth of the test interference filters at 370, 410, 490, and 570 nm.

These filters are close to the absorbance peaks of adult zebrafish UV, blue, green, and red cones, respectively. Wavelengths of these filters are maximally absorbed by the opsins in these cones. The next most efficiently absorbing cone ranges from 4% (red vs green cones) to as much as 50% (UV vs blue cones).

filter	Min wavelength filter	Max wavelength filter	UV cone absorbance integral	Blue cone absorbance integral	Green cone absorbance integral	Red cone absorbance integral
370	360	380	0.90267	0.48965	0.15440	0.21177
410	400	420	0.06622	0.98901	0.31186	0.15894
490	480	500	0.00001	0.05323	0.94776	0.33159
570	560	580	0.00000	0.00002	0.04187	1.00116

Disease Models & Mechanisms • Supplementary information

Figure S1. Changes in glucose vs. mannitol native ERG parameters with a white light stimulation across all stimulus intensities.

Under 'native ERG' conditions, a-, b-, and d-waves are evoked in all treatments, and under most stimulus conditions. As with the previous analyses (Figures 2-4), glucose- and mannitol-treated ERG measures shown here are normalized to mean water control values. In the cumulative glucose/mannitol datasets (which represents means of 1546-2100 points, from 10 eyes per treatment, collected over 7 irradiances from 6.0-3.0 log units of beam attenuation with white (Xenon) stimuli on IR background), (A) a-wave amplitude in glucose-treated retinas was increased compared to mannitol; whereas (B) b-wave and (C) d-wave amplitudes in glucose-treated retinas were reduced. Glucose treatment quickened a-wave, b-wave and d-wave peak times (D, E, F). Water-treated mean (± s.e.m.) control values are (A) -1.10 ± 0.05 µV, n = 1477 responses; (B) 2.18 ± 0.10 µV, n = 1477; (C) 0.87 ± 0.05 µV, n = 1477; (D) 44.7 ± 0.51 ms, n = 1333; (E) 136.1 ± 1.03 ms, n = 1369; (F) 135.9 ± 2.09 ms, n = 1218. Asterisks use GraphPad significance convention ($p > 0.05$, *; $p \leq 0.05$, **; $p \leq 0.01$, ***; $p \leq 0.001$; ****, $p \leq 0.0001$). Amplitudes in each individual response are measured as mean voltages within intervals characteristic for the component: a-wave amplitude: 15-35 ms post onset, peak time, 10-100 ms; b-waves: 51-200 ms post onset; d-waves: 25-250 ms post offset. If a- b- and d-waves lacked maxima in the intervals specified, no peak time was scored, often the case with threshold stimulation, or severely affected eyes.

Disease Models & Mechanisms • Supplementary information

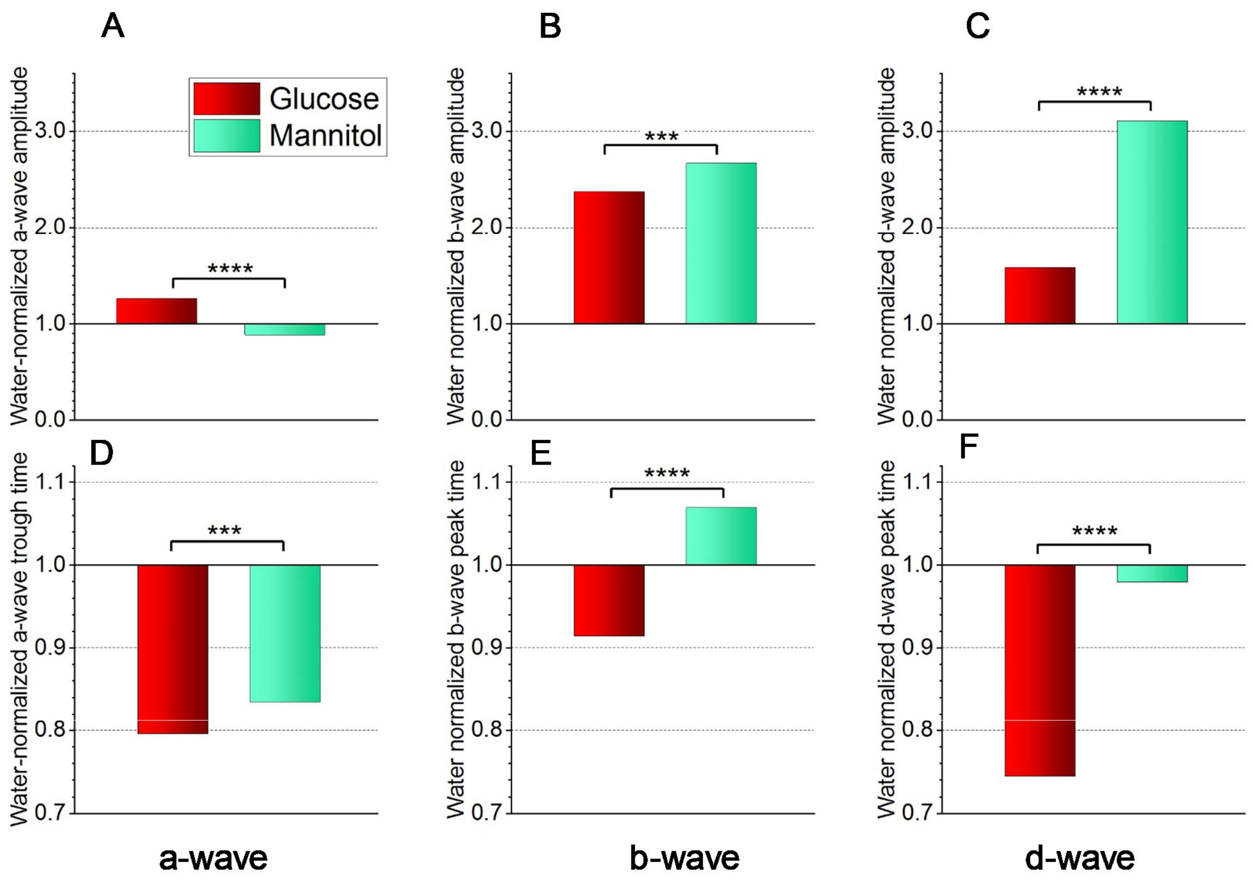

Disease Models & Mechanisms • Supplementary information

Figure S2. Cumulative dataset spectral ERG signals from outer retina.

Amplitudes and peak latencies recorded with either (A-D) a blue (418 nm) or (E-H) red (627 nm) background. b2-wave and a1-wave amplitudes are isolated with an AMPA/KA antagonist (CNQX, 50 µM), which blocks horizontal cell, OFF-bipolar cell, and inner retinal responses. The analysis includes responses to all 9 wavelengths and 7 irradiances used in the spectral protocol, ranging from 650-330 nm, and 2.24-6.69 log(quanta·μm^{-2}·s^{-1}). Waves are measured as mean amplitudes in characteristic time intervals, and peak times are considered valid only if occurring in those intervals (a1 mean amplitude, 10-65 msec; a1 peak time, 10-100 msec; b2 mean amplitude or peak time, 51-200 msec). Mean cumulative response amplitudes of b2- (A, E) and a1- (B, F) components; (C,G) mean b2 implicit time, (D,H) mean a1 implicit time. Glucose- and mannitol responses were normalized to mean cumulative water control values prior to analysis. On both adapting backgrounds, glucose-treated fish show reduced a1 and b2 amplitudes compared to mannitol treatments. Asterisks denote significant differences (*t*-test, GraphPad asterisk convention. ns = non-significant, α = 0.05). For (A, B), glucose a1 and b2 amplitudes are means from 1120 responses (9 eyes); mannitol, 1540 responses (13 eyes). (C) Glucose b2 peak times are means of 952 peaks (9 eyes); mannitol, 1323 peaks (13 eyes). (D) Glucose a1 trough times are means of 960 points (9 eyes); mannitol, 1365 points (13 eyes). On the blue background (418 nm, A-D), the water control values used for normalization (630 responses, 6 eyes) are: b2 amplitude, 7.49 ± 0.38 µV; a1 amplitude, -2.38 ± 0.12 µV, b2 peak time, 142.5 ± 1.4 ms, n = 534; a1 peak time, 59.8 ± 0.7 ms, n=579. For the red background (627 nm, E-H), glucose a1 and b2 amplitudes (E, F) are means of 1190 responses (9 eyes); mannitol responses are means of 1750 responses (13 eyes). (G) Glucose b2 peak times are means of 964 valid peaks (9 eyes); mannitol, 1526 peaks (13 eyes). (H) Glucose a1 trough times are means of 959 valid peaks (9 eyes); mannitol, 1453 peaks (13 eyes). The water control a1 and b2 amplitudes used for normalization (910 responses, 7 eyes) are b2 amplitude, 4.17 ± 0.25 µV; a1 amplitude, -2.63 ± 0.13 µV, b2 peak time, 154.4 ± 1.0 ms, n = 707; a1 peak time, 68.9 ± 0.5 ms.

Disease Models & Mechanisms • Supplementary information

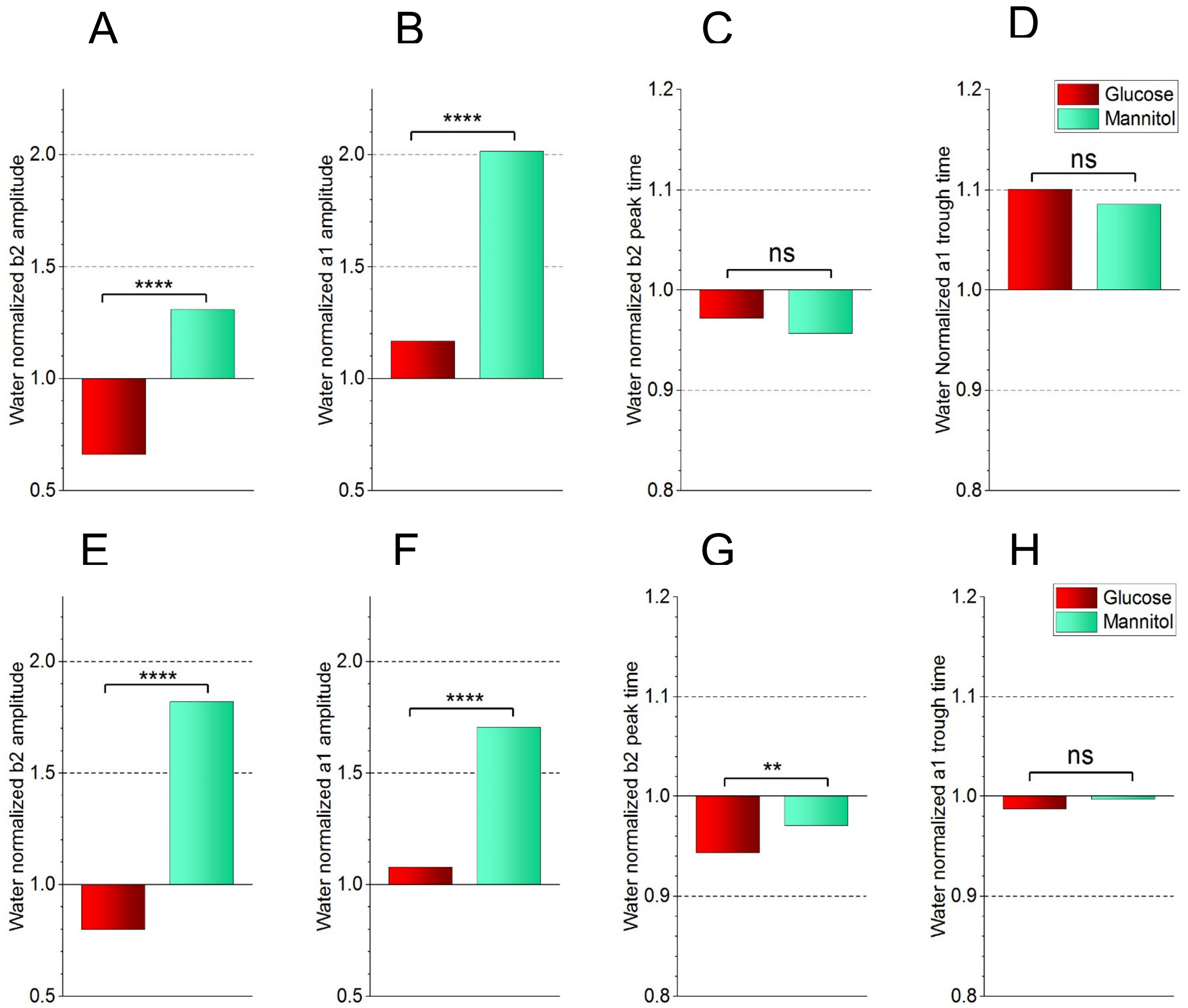
A
B
C
D
E
F
G
H
Water normalized b2 amplitude
Water normalized a1 amplitude
Water normalized b2 peak time
Water Normalized a1 trough time
Water normalized a1 trough time
Glucose
Mannitol
****
**
ns
0.5
1.0
1.5
2.0
0.8
0.9
1.1
1.2

Figure S3. Amplitude histogram for glucose and mannitol for 3.0 ND white light.
The tick mark denotes -11 µV in both cases. This histogram shows that the distribution of mannitol-treated b-wave amplitudes is much tighter than glucose-treated b-wave amplitudes. It also shows that mannitol treated amplitudes are all positive while some of the glucose-treated eyes were negative. In each case the b-wave amplitudes were the mean amplitudes in the interval 51-200 msec after the flash. Each distribution contains 300 amplitude measures on 10 eyes.

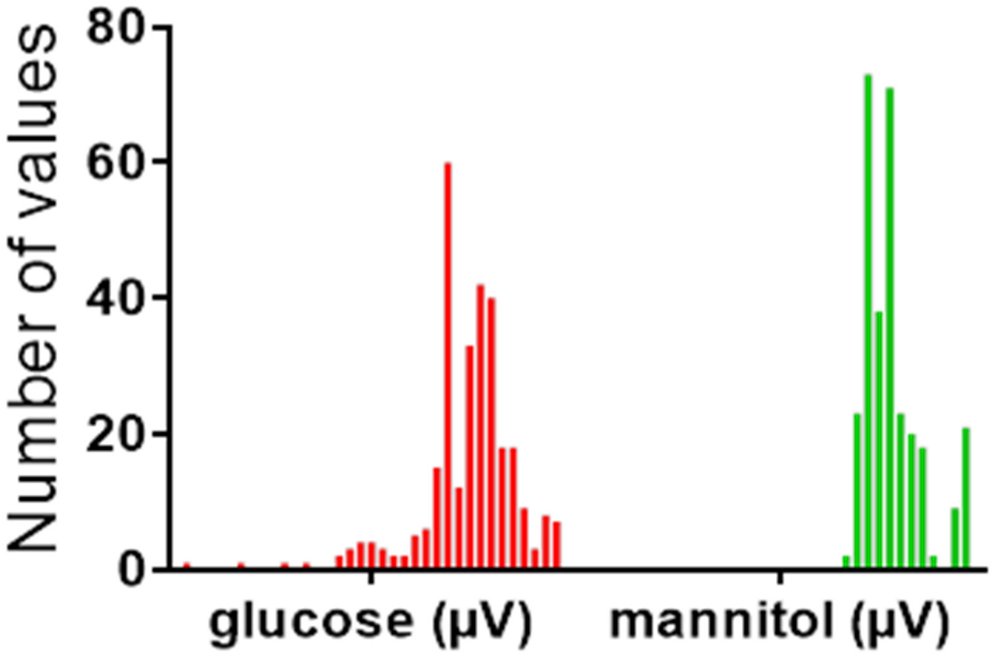

Supplement: Supplementary information [file dmm-11-035220-s1.pdf]
